# Heterogeneity in response to treatment across tinnitus phenotypes

**DOI:** 10.1038/s41598-024-52651-x

**Published:** 2024-01-24

**Authors:** Uli Niemann, Benjamin Boecking, Petra Brueggemann, Myra Spiliopoulou, Birgit Mazurek

**Affiliations:** 1 University Library, Otto von Guericke University Magdeburg, Universitätsplatz 2, Magdeburg, 39106 Germany; 2 Faculty of Computer Science, Otto von Guericke University Magdeburg, Universitätsplatz 2, Magdeburg, 39106 Germany; 3 Charité-Universitaetsmedizin Berlin, Corporate Member of Freie Universität Berlin and Humboldt-Universität zu Berlin, Charitéplatz 1, Berlin, 10117 Germany

**Keywords:** Diseases, Signs and symptoms

## Abstract

The clinical heterogeneity of chronic tinnitus poses major challenges to patient management and prompts the identification of distinct patient subgroups (or phenotypes) that respond more predictable to a particular treatment. We model heterogeneity in treatment response among phenotypes of tinnitus patients concerning their change in self-reported health burden, psychological characteristics, and tinnitus characteristics. Before and after a 7-day multimodal treatment, 989 tinnitus patients completed 14 assessment questionnaires, from which 64 variables measured general tinnitus characteristics, quality of life, pain experiences, somatic expressions, affective symptoms, tinnitus-related distress, internal resources, and perceived stress. Our approach encompasses mechanisms for patient phenotyping, visualizations of the phenotypes and their change with treatment in a projected space, and the extraction of patient subgroups based on their change with treatment. On average, all four distinct phenotypes identified at the pre-intervention baseline showed improved values for nearly all the considered variables following the intervention. However, a considerable intra-phenotype heterogeneity was noted. Five clusters of change reflected variations in the observed improvements among individuals. These patterns of treatment effects were identified to be associated with baseline phenotypes. Our exploratory approach establishes a groundwork for future studies incorporating control groups to pinpoint patient subgroups that are more likely to benefit from specific treatments. This strategy not only has the potential to advance personalized medicine but can also be extended to a broader spectrum of patients with various chronic conditions.

## Introduction

Tinnitus is the perception of a phantom sound when no corresponding external sound is present. It is estimated that approximately 15% of the adult population is affected^1^. Severe tinnitus (1–2%^2^) is associated with a considerable impairment in quality of life^2^ and an immense socioeconomic burden^3^. Because tinnitus is a symptom of possibly multiple underlying conditions and different accompanying comorbidities^2^, a detailed medical examination and assessment of the clinical history of the tinnitus patient is pivotal^4,5^. Clinical evaluation is challenging due to patient heterogeneity in several factors, including tinnitus perception (e.g. laterality, pitch, noise characteristics, frequency, duration, chronicity), risk factors (including hearing loss, age), comorbidities (including hyperacusis, depression, sleep disorders), perceived distress, predisposing psychological state, and response to treatment^6^.

To date, there is no tinnitus treatment that is effective for all patients, which has been often attributed to tinnitus heterogeneity^2,6,7^. Therefore, much of the research is devoted to identifying tinnitus subtypes, hereafter denoted as *phenotypes*, that respond more homogeneously, and thus more predictably, to a particular treatment^6,8,9^. Knowledge of such phenotypes could allow more targeted treatment selection or the development of more effective therapies^6^. As clinically relevant phenotypes have not been established yet and in the absence of a ground truth, there are several works on machine learning approaches^10^. However, no detailed study has examined how phenotypes change with treatment and whether phenotypes are predictive of the extent of response to treatment.

Our aim was to model and explore heterogeneity in response to a seven-day multimodal treatment among tinnitus patients in a large cohort from Germany by visualizing their changes between pre- and post-intervention assessments with respect to self-reported health burden, psychological characteristics, and tinnitus characteristics.

## Methods

### Study participants and treatment

The analyses were based on data from patients with chronic subjective tinnitus treated at the Tinnitus Center of Charité Universitätsmedizin Berlin between January 2011 and October 2015. All patients had suffered from tinnitus for three months or longer and were at least 18 years old. Exclusion criteria were acute psychotic illness or addiction, deafness, and insufficient knowledge of the German language. Patients who had objective tinnitus were excluded from the present therapy and treated with medication if necessary.

Patients underwent a comprehensive seven-day multimodal intervention program integrating three primary approaches. Firstly, there was a psychological intervention, centering on Cognitive Behavioral Therapy (CBT), conducted daily in group sessions and three times in personalized individual settings. The second focus involved listening exercises aimed at guiding perception, reducing avoidance, and fostering mindfulness within the listening room. These exercises took place daily in group sessions and through individual homework training. The third focus was on body-related procedures, encompassing daily muscular relaxation exercises within group settings and 3–4 individual physiotherapy sessions. These daily interventions were complemented by counseling from medical professionals during admission, discharge, and daily ward rounds. Furthermore, psychologists and audiology specialists were available throughout the day to address any patient queries. Two concentrated information sessions were delivered through lectures. The program also included psychoeducation sessions designed to tackle sleep difficulties and explore the intricate connections between hearing and emotional stimulus processing. Admission diagnostics were conducted on the first day of inpatient therapy ($t_0$) using a tablet. On the day of discharge ($t_1$), post-treatment diagnostics were conducted using the same tools, immediately before the patient left the program.

Ethical approval was granted by the ethics committee of Charité-Universitätsmedizin Berlin (EA1/115/15). Informed written consent was received from all patients. All relevant guidelines and regulations have been followed, including the Declaration of Helsinki. Prior to the analyses, all data were pseudonymized.

### Selected variables

Patients completed a series of routine questionnaires at the pre-treatment baseline $t_0$ and post-treatment $t_1$. The questionnaires were selected to obtain a comprehensive assessment of tinnitus, including tinnitus-related complaints and the psychosomatic background of tinnitus with anxiety, depression, general quality of life, and experienced physical impairments. For data analysis, a total of 64 variables from 14 questionnaires were used, including 49 composite scores and 15 single-item measures. The variables were grouped into ten categories:

1. *Tinnitus characteristics (11 variables):*
  (3) The visual analogue scales [TINSKAL] loudness, frequency, and distress
  (8) Binary variables for tinnitus localization (left or right ear, both ears, or entire head) and noise (whistling, hissing, ringing, rustling) from the *Tinnitus Localization and Quality Questionnaire*^11^ [TLQ]
2. *Physical quality of life (4):* The scores overall health, physical component, physical functioning, and role emotional from the *Short Form-8 Health Survey*^12^ [SF8]
3. *Experiences of pain (7):*
  (2) The scores affective pain and sensoric pain from the *Pain Perception Scale*^13^ [SES]
  (1) The SF8 bodily health compound score
  (4) Visual analogue scales [SSKAL] for pain impairment, frequency, and intensity
4. *Somatic expressions (5):* The scores exhaustion, abdominal symptoms, limb pain, heart symptoms, and overall complaints from the *Berlin Complaint Inventory (Berliner Beschwerdeinventar*^14^ [BI]
5. *Affective symptoms (16):*
  (1) The total score from the *General Depression Scale (Allgemeine Depressionsskala)*^15,16^ [ADSL]
  (6) The scores fatigue, apathy, anxious depression, anger, positive mindset, and elevated mood from the *Berlin Mood Questionnaire (Berliner Stimmungsfragebogen)*^17^ [BSF]
  (7) The subscales depressive syndrome, anxiety syndrome, obsessive-compulsive syndrome, somatoform syndrom, eating disorder syndrome, additional items, and the total psychiatric syndrom score from the *ICD-10 Symptom Rating*^18^ [ISR]
  (2) The depressivity score and the binary variable panic syndrome from the *(short form) Patient Health Questionnaire*^19^ [PHQK]
6. *Tinnitus-related distress (8):* The scores auditory perceptual difficulties, cognitive distress, emotional distress, intrusiveness, psychological distress, sleep disturbances, somatic complaints, and the total tinnitus distress score from the German version of the *Tinnitus Questionnaire*^20^ [TQ]
7. *Internal resources (3):* The scores self-efficacy, optimism, and pessimism from the *Self-Efficacy- Optimism-Pessimism Scale questionnaire (Selbstwirksamkeits-Optimismus-Pessimismus Skala)*^21^ [SWOP]
8. *Perceived stress (5):* The scores demand, tension, joy, worries, total perceived stress from the *Perceived Stress Questionnaire*^22^ [PSQ]
9. *Mental quality of life (6):*
  (1) The *Anamnestic Comparative Self-Assessment)*^23^ [ACSA] visual analogue scale on general quality of life.
  (5) The scores mental health, role emotional, social functioning, vitality, and mental component from SF8

In addition, the patients’ age, gender, education level, marital status, partnership status, duration of tinnitus, number of physicians visited in the past 12 months, and duration of ongoing psychological treatment were recorded.

### Data processing

Of a total of 4103 patients, 1228 completed all questionnaires at $t_0$ of which 989 patients completed all questionnaires also at $t_1$ (Supplementary-A). Unless otherwise indicated, the 989 patients with complete data at $t_0$ and $t_1$ were used. The coding of variables where higher scores indicate lower symptom burden was reversed (new value = maximum value − old value) to standardize the interpretation that higher scores mean higher disease burden. Variable names with a *-suffix denote reversed variables. Because of the varying ranges of values, each variable was normalized to a mean of 0 and a standard deviation of 1 using z-score normalization prior to data analysis.

### Baseline phenotypes

In our previous work^24^, we identified four phenotypes of tinnitus patients at $t_0$ using a non-parametric clustering algorithm that automatically determines the appropriate number of subgroups^25^. Phenotype 1 (PT 1), labeled “avoidant group”, forms the largest subgroup (697 of 1228 patients; 56.8%) and is characterized by substantially below-average symptom expression on tinnitus-related and more general psychosomatic symptom indices, including affective symptoms, perceived stress, tinnitus-related distress, and somatic symptoms, as well as (above-average) quality of life and internal resources. Phenotype 2 (PT 2,“psychosomatic group”) is the second smallest subgroup (173 patients; 14.1%), and the phenotype with the highest emotional and somatic burden, showing a high tinnitus burden in addition to clinically relevant impairment in all affective indices, including depression, anxiety, and perceived stress. Furthermore, patient of this phenotype reported highest somatoform expressions of distress, including somatic symptoms. Phenotype 3 (PT 3; “somatic group”) is the second largest subgroup (187 patients; 15.2%) and characterized by above-average scores measuring somatic complaints and near-average scores for affective symptoms. Phenotype 4 (PT 4; “distress group”) is the smallest subgroup (171 patients; 13.9%), has above-average values for affective scores, quality-of-life components, and perceived stress.

### Modeling static patient phenotypes in a reduced variable space

We induced patient phenotypes in a projected variable space at $t_0$. We introduced dimensionality reduction to concentrate only on the variance-reducing features when monitoring phenotype evolution. We chose Uniform Manifold Approximation and Projection (UMAP)^26^, a dimensionality reduction technique used in machine learning and data visualization. UMAP combines a graph-based approach to capture local relationships with an optimization process that balances the preservation of both local and global structures within high-dimensional data. Unlike linear methods such as Principal Component Analysis (PCA)^27^, UMAP is capable of revealing non-linear relationships, offering enhanced flexibility in exploring intricate data structures. UMAP is widely used in communities dealing with high-dimensional data, such as population genetics^28^.

In addition to showing patients in projected two-dimensional space, we designed a visualization inspired by PCA factor maps, where each variable is depicted as an arrow. The direction of a vector, i.e., its angle, shows the direction of correlation with the projected dimensions. For example, an arrow pointing to the right indicates a positive correlation with the x-axis, whereas a downward arrow indicates a negative correlation with the y-axis. Also, the more parallel a vector is to an axis, the more strongly it is correlated with that projected dimension only. The length of a vector can be interpreted as the strength of the correlation. The longer the arrow, the stronger the correlation with one or both of the projected dimensions. The angles between vectors of different features show their correlation in the projected space. For example, an angle of 0° represents a high positive correlation, an angle of 90° indicates a lack of correlation, and an angle of 180° indicates a high negative correlation.

### Modeling the phenotypes’ change with treatment

The *medoid* is a representative patient of a phenotype whose sum of Euclidean distances to all patients of the phenotype is minimal. The change in coordinates of a medoid over time shows the average effect of treatment for patients of a phenotype. The direction of change can be interpreted with respect to the original dimensions using the factor map. By comparing the vectors spanning the medoid coordinates of successive time points, differences in treatment efficacy between phenotypes can be detected. The *core hull* of a phenotype *i* is the convex hull of patients in *i* whose distance to the medoid of *i* is smaller equal the median of the distances of all patients in *i* and the medoid of *i*, in other words, the 50% of patients closest to the medoid. If the area of the core hull increases, this indicates large differences in the effectiveness of the treatment for the patients within the same phenotype. After computing the medoid and the core hull of a phenotype at $t_0$, we assigned the patient data at $t_1$ to the cluster with the most proximal medoid. Thereafter, we computed the core hull at $t_1$ and use a Sankey diagram to visualize the “transition” of patients between phenotypes from $t_0$ to $t_1$.

### Determination of pathways of change

We determined “pathways of change” (PC) by performing cluster analysis^25^ on the differences between the variables’ values in $t_1$ and $t_0$, denoted as $\Delta (t_0,t_1)$. Hence, negative values $\Delta (t_0,t_1)$ indicate an improvement, signifying that the $t_1$ score is smaller than the $t_0$ score. To understand whether $t_0$ features are associated with the PC, we computed the Spearman correlation for each $t_0$ feature and each PC. Finally, we juxtaposed the relationship of phenotypes and pathways of change with a mosaic chart.

## Results

### Phenotypes in $t_0$

**Figure 1:**
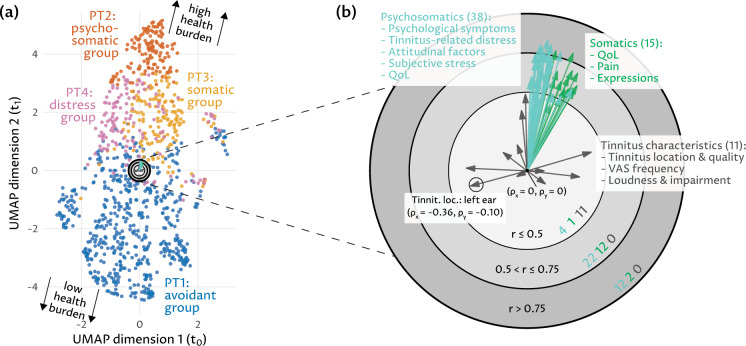
Two-dimensional (UMAP) projection of tinnitus patients ($t_0$) and correlation of original variables with projected dimensions. (**a**) Result of UMAP dimension reduction where each point depicts a patient. Points that are close to each other represent patients that are similar in the original 64D space. (**b**) Factor map indicating the relation between the original variables and the projected dimensions. The rings of the factor map summarize three gradations of the strength of the linear correlation between the original variables and the UMAP dimensions, while the rotated labels indicate the number of variables per group.

As reported in our previous study^24^, four phenotypes were identified on the pre-treatment data ($t_0$) that showed distinctive differences in symptom expression on tinnitus-related and more general psychosomatic symptom indices, affective symptoms, perceived stress, tinnitus-related distress, somatic symptoms, quality of life, and internal resources (Supplementary-B). In the UMAP projection (Fig. 1a), the transitions between the phenotypes are gradual. The second UMAP dimension shows higher variability than the first, as many original variables are correlated with the second UMAP dimension (Fig. 1b). From 38 psychosomatic variables in total, 12 exhibit a high correlation (r $\ge$ 0.75) with the projected dimensions, including compound scores measuring depressivity, stress, tinnitus distress, fatigue, additional items, tension, and worries. Further, 2 out of 15 somatic compound scores are also part of the outer ring, i.e., the overall complaints and fatigue scores. The tinnitus characteristics on tinnitus location and quality exhibit low correlation to the projected dimensions and to the psychosomatic and somatic variables, respectively (Fig. 1b).

**Figure 2:**
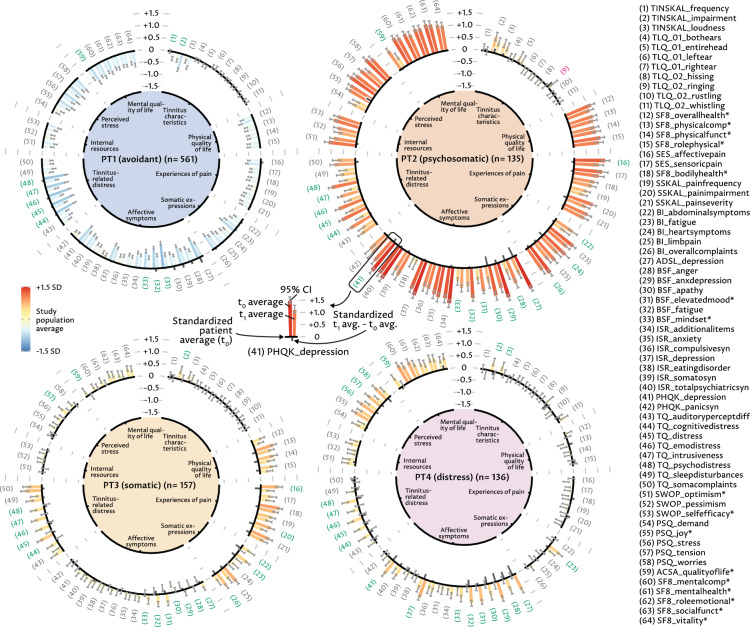
Within-phenotypes averages for each variable in $t_0$ and $t_1$. Radial bar graphs for each phenotype showing standardized mean within-phenotype variable averages. Outward-facing (inward-facing) colored bars represent variable averages that are above (below) the study population average. For each variable, two bars are shown, for $t_0$ and $t_1$ (arranged in clockwise order). Variable labels are colored in green if the $t_0$ score exceeds the $t_1$ score by more than 0.25 and red if the $t_1$ score exceeds the $t_0$ score by more than 0.25. The black bars in between pairs of colored bars depict the standardized difference between the phenotype average in $t_0$ and $t_1$, where inward (outward) facing bars represent variables where the phenotype patients improved, on average, more (less) than the general study population.

### Change of phenotypes with treatment

On average, all phenotypes showed improved values for nearly all the considered variables following the multimodal treatment (Fig. 2, Supplementary-B). For PT1 and PT3, a score that measures elevated mood improved most. For PT2 and PT4, a variable on depression reduced most between $t_0$ and $t_1$. Among the top ten variables with the highest improvement among all phenotypes were variables on elevated mood, cognitive distress, psychological distress, emotional distress, tinnitus distress and fatigue. The benefit lies in changing variables that maintain psychological distress and which are addressed by psychological therapy. As such, results are in line with research that pinpoints psychological treatment approaches as gold standard for treating chronic tinnitus^29^. PT2 showed highest above-average improvement in depression and apathy. PT3 showed above-average improvement in several somatic scores (cf. categories physical quality of life, experiences of pain, and somatic expressions). PT4 (distress group) exhibited mainly above-average reduction in affective symptoms scores measuring depression, anger, apathy, and fatigue.

**Figure 3:**
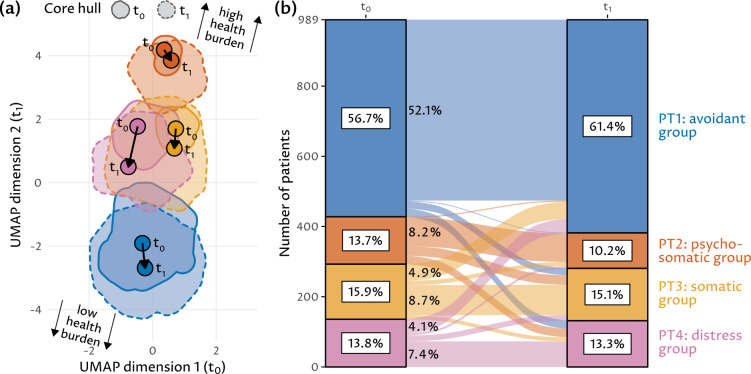
Visual representation of (**a**) the change of each phenotype’s medoid and core hull from $t_0$ to $t_1$, and (**b**) patient transition between phenotypes from $t_0$ to $t_1$. (**a**) UMAP projection where each point symbolizes the phenotype medoid enclosed by the convex hull of the core ($t_0$: solid line; $t_1$: dashed line). (**b**) A patient’s phenotype membership is determined by the medoid with the smallest Euclidean distance. The relative size of each phenotype is indicated in the text boxes; the relative number of patients changing from $t_0$ to $t_1$ to a different phenotype are shown on the left side of the curves, with only subgroups above 3% shown.

Figure 3a visualizes the change of each phenotype’s medoid and core hull from $t_0$ and $t_1$. On average, all phenotype appear to benefit from treatment, as the positions of all medoids move towards the area of low distress burden (bottom-left). Further, the increasing core radii of especially PT2, PT3, and PT4 indicate substantial within-phenotype heterogeneity in treatment efficacy. Figure 3b depicts treatment-induced patient transition between phenotypes. While for each phenotype the majority of patients remain in the same phenotype in $t_1$, the number of patients that are closest to PT1 increases from $t_0$ to $t_1$, as many patients from PT3 and PT4 move towards PT1 which also grew in size, reflecting the observed improvements in variable scores across all clusters.

### Pathways of change

**Figure 4:**
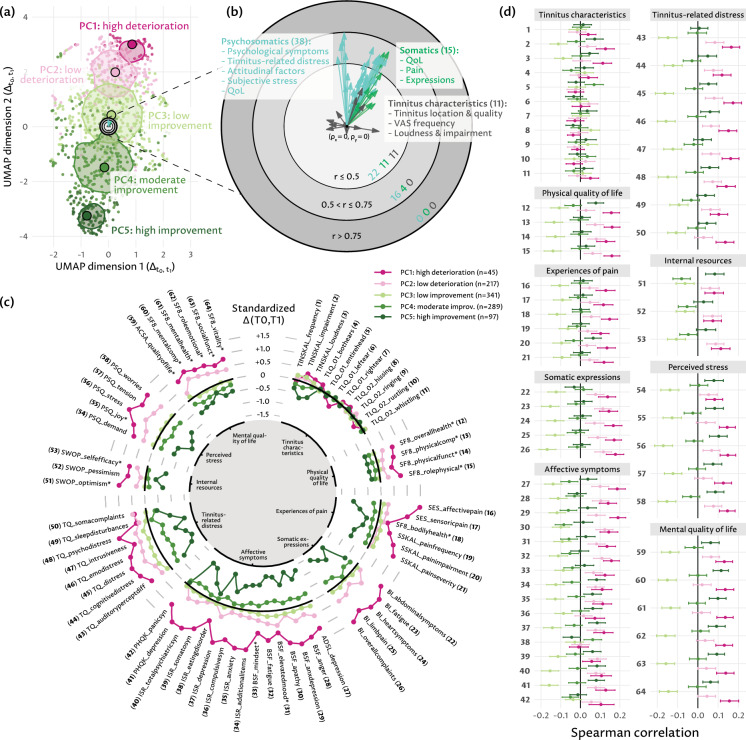
Two-dimensional (UMAP) projection of $\Delta (t_0,t_1)$ pathways of change, correlation of original variables to projected dimensions, comparison of pathways of change, and correlation of original variables to pathways of change. (**a**) Result of UMAP dimension reduction on $\Delta (t_0,t_1)$. Each point symbolizes a patient. Points that are close to each other represent patients that are similar with respect to $\Delta (t_0,t_1)$ in the original 64D space. (**b**) Factor map indicating the relationship between the original variables and the projected dimensions. (**c**) Radial line graph for the comparison of pathways of change (PC). Points show within PC variable averages. Points depicting variables of the same category are connected with line segments. Points and lines are colored by PC. (**d**) Spearman correlation of each original variable with each PC. The 95% confidence intervals were estimated by bootstrap sampling using 2000 samples and the percentile method^30^.

Five *pathways of change* (PCs) were identified, representing the alterations in variable scores between $t_0$ and $t_1$, relative to the study population. These subgroups were named as follows (Fig. 4a): *PC1: high deterioration* (45 individuals, 4.6%), *PC2: low deterioration* (217 individuals, 21.9%), *PC3: low improvement* (341 individuals, 34.5%), *PC4: moderate improvement* (289 individuals, 29.2%), and *PC5: high improvement* (97 individuals, 9.8%). Supplementary-Cprovides a summary of the average values for $t_0$, $t_1$, and $\Delta (t_0,t_1)$ for each PC. The factor map in Fig. 4b shows that 16 psychosomatic and 4 somatic $\Delta (t_0,t_1)$ variables exhibit a moderate to high correlation to the UMAP projection (cf. middle ring), whereas most variables from all three groups have a low correlation to the UMAP projection (cf. inner ring (r $\le$ 0.5). Most psychosomatic and somatic variables are positively correlated with the second UMAP dimension (cf. arrows pointing upwards) and some of them are positively correlated with the first UMAP dimension (cf. arrows pointing to the right), meaning that patients with less score improvements are more likely to be seen in the top and top-right of the projection. The radial line graph in Fig. 4c shows standardized within-PC averages for each $\Delta (t_0,t_1)$ variable. The PC are particularly well separated for all variables except the tinnitus characteristics. PC1 is exclusively and PC2 is predominantly above the mean of the study population for these eight categories, whereas PC3 is mostly above and PC4 and PC5 are exclusively below the mean of the study population for these eight categories. Figure 4d reveals no or negligible correlation of original variables ($t_0$) to the PC, indicating no predictiveness of the $t_0$ variables to $\Delta (t_0,t_1)$ as a weak correlation is typically defined between 0.2 and 0.3.

### Association of phenotypes with response to treatment

**Figure 5:**
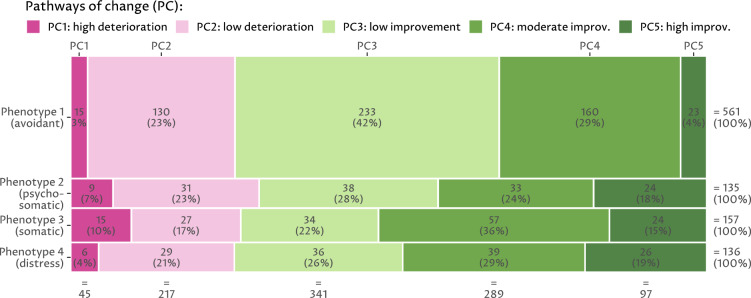
Juxtaposition of the four phenotypes with the five pathways of change. Mosaic plot showing the number of patients for each combination of phenotype (PT) and pathway of change (PC). The size of each area is proportional to the number of patients in each segment. Percentages show a segment’s size relative to the number of patients with the associated phenotype.

The proportion of subjects in each PC differed by phenotype, $X^2 (df = 12, N = 989) = 84.145, p <0.05$. Figure 5 shows the number of patients for each combination of phenotypes and pathways of change, with the size of the boxes proportional to the number of patients. About 75% of the patients are associated with PC 3–5 which exhibited improved variable scores, with the two negative PC accounting for only about 25%. Compared with the other phenotypes, PT1 includes a lower proportion of patients with the more extreme PC1 and PC5. PT3 includes a higher proportion of patients with moderately or highly positive variable score changes, but also slightly more patients with the strongly negative PC 1. Compared to PT1, PT2 has a higher proportion of patients whose condition actually worsens.

## Discussion

On average, each phenotype showed mainly improved variables scores after the multimodal treatment program that aimed to relieve distress attributed to chronic tinnitus, confirming the effectiveness of psychologically anchored approaches as gold standard for alleviating tinnitus-related distress^5,31^. The findings both confirm and expand previous findings on the effectiveness of the here-investigated intensive, multimodal seven-day program. Previous studies demonstrated effectiveness for 3-, and 5-year follow-up timepoints^32,33^, and the treatment appears to be beneficial for the majority of patients. Beyond this finding, results from the present study demonstrate that (1) subgroup characteristics of chronic tinnitus patients at baseline do not map onto (2) subgroup characteristics of chronic tinnitus patients that benefit from the psychologically anchored, multimodal, intensive short-term treatment approach. Future studies should therefore

- delineate between whether they aim to (1) describe the chronic tinnitus-population or (2) predict responsiveness to a psychological treatment approach (for which other sets of predictors apply), and
- map measured variables on theories of psychosomatic theories to chronic tinnitus (1) onset, (2) maintenance, and (3) respective treatment focus.

Emotional distress, as defined by the Tinnitus Questionnaire (TQ), and emotional avoidance, hypothesized to underlie Phenotype 2, did not appear to predict treatment response. Future studies should explicitly operationalize and measure these psychological process variables to predict treatment response or chronic tinnitus symptomatology at baseline, respectively.

Overall, results demonstrated large within-phenotype heterogeneity in treatment response. Across phenotypes, i.e., irrespective of health burden, psychological characteristics (distress indicators, attitudinal factors, subjective stress, quality of life, pain experience and additional somatic distress expressions) were highly intercorrelated and largely independent from tinnitus characteristics (localization, sound, etc.). The finding echoes previous investigations on the independence of tinnitus characteristics and subjective distress^34^. It further underscores the importance of (partially pre-existing) affective factors that influence the processing of the tinnitus stimulus and thereby potentially facilitating a trajectory towards “tinnitus disorder”^35–43^.

Whilst all phenotypes appear to benefit from treatment, the distress group, characterized by overall medium (mental) health burden, exhibited most improved variable scores, whilst similarly burdened patients who expressed distress in a more somatic manner exhibited the least improved scores after the here-examined treatment. Reasons are speculative, yet may comprise the explicit distress focus of the treatment program as well as roles of psychological factors that may hinder treatment success in a short-term setting^44^.

Because baseline phenotypes did not predict treatment response, it becomes crucial to identify and measure psychological dimensions that may better serve as predictors of treatment responsiveness. In the context of the here-observed psychologically anchored treatment program, such variables could include exemplary psychological variables from the so-called “common factors literature” such as “insight into existing difficulties”, “difficulties identifying and describing feelings”, “emotional avoidance”, “subjective experiences of therapeutic alliance” or “subjectively felt match between individual difficulties and offered treatment components and -foci”. The large variance in the psychological treatment-response profiles suggests a need for applying psychological assessment and treatment frameworks that consider relational as well as experience-based variables in explaining treatment response. Outpatient assessment or -treatment contacts prior to commencing the multimodal outpatient treatment program may offer additional sources to operationalize meaningful phenotype-definition criteria in future research^45,46^. Overall, research on treatment-response phenotype might benefit from linking phenotype-constituting variables with theory and practice of the respectively examined treatment options.

Treatment effect patterns did reveal significant, but not clinically meaningful differences in pathway-of-change-proportions across baseline phenotypes, again highlighting the need to distinguish between baseline and treatment-success-related phenotypes, particularly when advancing towards personalized medicine for a variety of chronic conditions^47–52^. A potential explanation for the rather small difference between variable changes in all baseline phenotypes, independent of the baseline phenotypes themselves, may be attributed to a symptom-focused measurement approach in this study. The psychological treatment applied in this study addressed factors known to sustain tinnitus-related distress rather than focusing explicitly on the symptoms. Future studies should consider defining phenotypes by grouping psychological maintaining factors associated with tinnitus-related distress^53,54^. This finding suggests that defining phenotype characteristics should align with evidence-based treatment approaches and their underlying mechanisms of change, emphasizing the need to move beyond surface-level symptoms.

As a consequence, mapping clinical phenotypes to intervention strategies becomes more meaningful without conflating “symptoms” and “maintaining mechanisms”. Individualized psychological treatment strategies can then precisely target the latter, providing a more nuanced and effective approach to addressing the complexities of chronic conditions.

Indeed, the heterogeneity in treatment effect among the baseline phenotypes is reflected in their partial dissolution (cf. increasing core hull areas in Fig. 3a). Our approach poses a pragmatic way to identify patient subgroups that are likely to benefit from a given treatment, offering a strategy that may facilitate personalized medicine^55,56^ and be applicable towards a large group of patient populations with medical or psychosomatic chronic conditions.

Several limitations should be acknowledged. The lack of control or waiting groups limits our ability to fully discern potential repetition effects between $t_0$ and $t_1$ measures. Future research incorporating such groups could provide a more robust understanding of treatment outcomes.

The uniformity in the delivery of therapy without accounting for variance in individual modules restricts our ability to isolate and evaluate the effects of specific therapeutic components. A more detailed exploration of individual therapy modules would enhance our comprehension of their respective impacts.

Our analysis focused on only a quarter of the total patient population, raising the possibility that individuals who were able to complete all questionnaires might not be fully representative of the broader patient demographic. Future studies should aim for a more comprehensive inclusion of participants.

The absence of demographic variables in our analysis limits our ability to assess potentially differing treatment effects for various demographic groups. Future research should systematically consider and analyze the impact of demographic factors on treatment outcomes.

The lack of information on comorbidities and prior psychiatric conditions hinders our ability to explore potential variations in treatment effects among patients with present or preceding mood or anxiety disorders. Including this information in future studies would offer a more nuanced understanding of treatment outcomes.

An intensive multimodal treatment approach presents a nuanced balance of advantages and drawbacks. In the short term, these intensive multimodal treatments emerge as the preferred choice for addressing somatoform and psychosomatic challenges, such as chronic tinnitus. They have demonstrated compelling efficacy, notably in chronic pain treatment^57^. Chronic pain and chronic tinnitus share some phenomenological overlap^58^. Patients undergoing this approach have the opportunity to comprehend chronic tinnitus through a variety of interconnected lenses—psychological, medical, and physiotherapeutic. Within this comprehensive biopsychosocial framework, the goal is for patients to develop a de-catastrophizing understanding of tinnitus and its psychological maintenance. Multimodal intervention strategies are designed to alleviate distress and, consequently, provide relief from symptoms. The structured group dynamic formed during the fixed treatment duration additionally fosters a supportive atmosphere. However, the brevity of the program limits the observation and training of explored behavior modifications. While there is ample time to introduce treatment modules and motivate patients to incorporate therapeutic elements into their daily lives, it may not suffice for in-depth treatment of potential comorbidities, such as psychiatric disorders within the depression spectrum. In such cases, referrals to specialists and potentially intensified treatment measures become necessary. Nevertheless, empirical evidence supports the efficacy of the program^32^, demonstrating long-term changes in multimodal tinnitus therapy during a 5-year follow-up. The program consistently yields small yet stable effects.

Finally, we chose UMAP for dimensionality reduction to visualize complex patterns in high-dimensional tinnitus patient data, aiming to uncover patient phenotypes and pathways of change in an unsupervised setting. UMAP, a nonlinear technique, offers flexibility for visualizing high-dimensional data in lower-dimensional spaces, crucial when linearity cannot be assumed. We justified our choice through a comprehensive comparison with four other algorithms, including principal component analysis and t-SNE. Results in Supplementary-D show similar projections across techniques. Notably, linear methods like PCA tend to emphasize outliers, reinforcing the suitability of UMAP for capturing complex, non-linear relationships in our data.

### Supplementary Information

Supplementary Information.

## Data Availability

As per the ethics committee of Charité-Universitätsmedizin Berlin, we cannot publish the data because we do not have the patients’ consent to publish their data. Interested researchers can contact the directorate of the Tinnitus Center of Charité-Universitätsmedizin Berlin with requests for data access: birgit.mazurek@charite.de.
