# Supplementary material for: Heterogeneity in response to treatment across tinnitus phenotypes
Source: Sci Rep. 2024 Jan 24;14:2111. doi: 10.1038/s41598-024-52651-x (PMC10808188; doi:10.1038/s41598-024-52651-x)
Supplement: Supplementary file 1 — Supplementary Information. [file 41598_2024_52651_MOESM1_ESM.pdf]

# – Supplementary Material –

Heterogeneity in response to treatment across tinnitus phenotypes

Uli Niemann[1,2,*], Benjamin Boecking[3], Petra Brueggemann[3], Myra Spiliopoulou[2], and Birgit Mazurek[3]

[1] *University Library, Otto von Guericke University Magdeburg, Universitätsplatz 2, Magdeburg 39106, Germany*
[2] *Faculty of Computer Science, Otto von Guericke University Magdeburg, Universitätsplatz 2, Magdeburg 39106, Germany*
[3] *Charité-Universitaetsmedizin Berlin, Corporate Member of Freie Universität Berlin and Humboldt-Universität zu Berlin, Charitéplatz 1, Berlin 10117, Germany*
[*] *Corresponding author; uli.niemann@ovgu.de*

Supplementary A: patient demographics

Of a total of 4103 patients (i), 1228 (ii) completed all questionnaires at t_0 of which 989 (iii) patients completed all questionnaires also at t_1. In our previous study,[1] we used the subset of 1228 patients with complete questionnaire data at t_0 to discover the four phenotypes. Because we investigated the phenotypes' change with treatment in the present article, we concentrated on the subset of 989 patients with complete questionnaire data at t_0 *and* t_1. The demographic characteristics and self-reported tinnitus distress of the study population and the two subsets are presented in Table 1.

	**All patients (i)**	**Patients with complete data at: t_0 (ii)**	**t_0 and t_1 (iii)**
Total (n)	4103	1228	989
Age (mean ± SD)	51 ± 13	50 ± 12	49 ± 12
Sex			
- Female	1849 (45%)	616 (50%)	490 (50%)
- Male	2034 (50%)	611 (50%)	498 (50%)
- Unknown	220 (5%)	1 (0%)	1 (0%)
Years since tinnitus onset			
≤ 0.5	796 (19%)	188 (15%)	154 (16%)
- 0.5 - 1	766 (19%)	264 (21%)	216 (22%)
- 1 - 2	573 (14%)	183 (15%)	151 (15%)
- 2 - 5	598 (15%)	186 (15%)	151 (15%)
- > 5	1150 (28%)	406 (33%)	316 (32%)
- Unknown	220 (5%)	1 (0%)	1 (0%)
Education level			
- Abitur/Fachabitur (higher education entrance qualification)	2,091 (51%)	730 (59%)	599 (61%)
- Mittlere Reife (≈ high school)	1193 (29%)	361 (29%)	280 (28%)
- Hauptschule (lower secondary education)	552 (13%)	128 (10%)	103 (10%)
- Other / unknown	267 (7%)	9 (1%)	7 (1%)
Family status			
- Married	2,086 (51%)	641 (52%)	523 (53%)
- Divorced	515 (13%)	185 (15%)	143 (14%)
- Unmarried	1164 (28%)	378 (31%)	307 (31%)
- Unknown	338 (8%)	24 (2%)	16 (2%)
TQ score at t_0 (mean ± SD)	38 ± 18	39 ± 17	39 ± 17

Data are n (%) unless specified otherwise.

TQ = German version of the Tinnitus questionnaire.[2]

Table 1: Patient demographics and self-reported tinnitus distress.

Supplementary B: mean values of the study population and each phenotype for each feature and changes with treatment

Tables 2 and 3 depict the mean values for each feature at baseline (t_0), after treatment (t_1), and the change with treatment (Δ_{t_0,t_1}). Higher values for $\bar{x}^{t_0}$ and $\bar{x}^{t_1}$ represent higher symptom burden. Positive values for $\bar{x}^{\Delta_{t_0,t_1}}$ describe an increase in symptom burden.

Table 2: Mean values (± standard deviations) of the study population for each feature at baseline (t_0), after treatment (t_1), and the change with treatment (Δ_{t_0,t_1}).

		Study population (n=989)		
Feature	Description	$\bar{x}^{t_0}$	$\bar{x}^{t_1}$	$\bar{x}^{\Delta_{t_0,t_1}}$
ACSA_qualityoflife*	Quality of life during the last 2 weeks	5.78 ± 2.6	4.93 ± 2.5	-0.85 ± 2.0
ADSL_depression	Depressive disorder sum score	18.06 ± 11.4	13.26 ± 10.7	-4.80 ± 8.2
BI_abdominalsymptoms	Abdominal symptoms score	3.09 ± 3.4	2.44 ± 3.3	-0.65 ± 2.5
BI_fatigue	Fatigue score	9.13 ± 5.7	7.99 ± 6.0	-1.15 ± 3.8
BI_heartsymptoms	Heart symptoms score	3.23 ± 3.7	2.81 ± 3.7	-0.43 ± 2.7
BI_limbpain	Limb pain score	8.84 ± 5.3	8.06 ± 5.5	-0.78 ± 3.3
BI_overallcomplaints	Overall complaints sum score	24.29 ± 15.0	21.30 ± 15.7	-3.00 ± 9.7
BSF_anger	Anger score	0.81 ± 0.7	0.61 ± 0.7	-0.20 ± 0.6
BSF_anxdepression	Anxious depression score	1.23 ± 0.9	0.93 ± 0.9	-0.30 ± 0.7
BSF_apathy	Apathy score	0.65 ± 0.7	0.46 ± 0.7	-0.19 ± 0.6
BSF_elevatedmood*	Elevated mood score	2.66 ± 0.9	2.16 ± 1.0	-0.50 ± 0.7
BSF_fatigue	Fatigue score	1.70 ± 1.0	1.29 ± 1.0	-0.41 ± 0.8
BSF_mindset*	Positive mindset score	1.94 ± 0.8	1.73 ± 0.8	-0.21 ± 0.6
ISR_additionalitems	Additional items score	0.74 ± 0.5	0.68 ± 0.6	-0.06 ± 0.4
ISR_anxiety	Anxiety score	0.93 ± 0.9	0.88 ± 0.9	-0.05 ± 0.7
ISR_compulsivesyn	Obsessive-compulsive syndrome score	0.78 ± 0.9	0.80 ± 0.8	0.02 ± 0.7
ISR_depression	Depression score	1.18 ± 0.9	1.03 ± 0.9	-0.15 ± 0.6
ISR_eatingdisorder	Eating disorder score	0.68 ± 0.8	0.64 ± 0.8	-0.04 ± 0.5
ISR_somatosyn	Somatoform syndrome score	0.61 ± 0.8	0.58 ± 0.8	-0.02 ± 0.6
ISR_totalpsychiatricsyn	Total psychiatric syndrome score	0.81 ± 0.6	0.76 ± 0.6	-0.05 ± 0.4
PHQK_depression	Presence of depression	8.45 ± 5.4	7.08 ± 5.4	-1.36 ± 3.7
PHQK_panicsyn	Presence of panic syndrome	0.09 ± 0.3	0.10 ± 0.3	0.01 ± 0.3
PSQ_demand	Demand score	0.50 ± 0.2	0.47 ± 0.2	-0.03 ± 0.1
PSQ_joy*	Joy score	0.52 ± 0.2	0.49 ± 0.2	-0.03 ± 0.1
PSQ_stress	Total perceived stress sum score	0.46 ± 0.2	0.43 ± 0.2	-0.03 ± 0.1
PSQ_tension	Tension score	0.59 ± 0.2	0.53 ± 0.2	-0.06 ± 0.1
PSQ_worries	Worries score	0.40 ± 0.2	0.36 ± 0.2	-0.04 ± 0.1
SES_affectivepain	Affective pain	24.26 ± 10.1	22.60 ± 9.6	-1.66 ± 7.1
SES_sensoricpain	Sensoric pain	13.69 ± 4.9	13.51 ± 5.0	-0.18 ± 4.0
SF8_bodilyhealth*	Bodily health score	13.01 ± 10.7	12.48 ± 10.4	-0.53 ± 7.4
SF8_mentalcomp*	Mental component summary score	25.78 ± 11.8	24.53 ± 12.1	-1.24 ± 7.9
SF8_mentalhealth*	Mental health score	15.23 ± 10.9	13.90 ± 10.8	-1.32 ± 8.1
SF8_overallhealth*	Overall health score	17.70 ± 7.0	16.86 ± 7.0	-0.83 ± 5.4
SF8_physicalcomp*	Physical component summary score	21.12 ± 9.8	20.88 ± 9.4	-0.24 ± 6.8
SF8_physicalfunct*	Physical functioning score	8.49 ± 8.2	8.41 ± 8.2	-0.08 ± 6.7
SF8_roleemotional*	Role emotional score	10.99 ± 9.6	10.52 ± 9.6	-0.47 ± 6.9
SF8_rolephysical*	Role physical score	9.15 ± 9.1	8.77 ± 8.8	-0.38 ± 7.5
SF8_socialfunct*	Social functioning score	9.37 ± 9.2	9.08 ± 9.1	-0.29 ± 7.4
SF8_vitality*	Vitality score	16.49 ± 7.7	16.05 ± 8.0	-0.44 ± 6.2
SSKAL_painfrequency	Visual analog scale pain frequency	4.61 ± 3.6	4.38 ± 3.6	-0.23 ± 3.2
SSKAL_painimpairment	Visual analog scale pain impairment	3.62 ± 2.9	3.37 ± 2.7	-0.25 ± 2.4
SSKAL_painseverity	Visual analog scale pain severity	2.82 ± 2.6	2.80 ± 2.6	-0.01 ± 2.1
SWOP_optimism*	Optimism score	1.27 ± 0.8	1.19 ± 0.8	-0.08 ± 0.5

Continued on next page

Feature	Description	Study population (n=989)		
		$\bar{x}^{t_0}$	$\bar{x}^{t_1}$	$\bar{x}^{\Delta_{t_0,t_1}}$
SWOP_pessimism	Pessimism score	2.11 ± 0.7	2.13 ± 0.7	0.02 ± 0.6
SWOP_selfefficacy*	Self-efficacy score	1.23 ± 0.6	1.16 ± 0.6	-0.07 ± 0.4
TINSKAL_frequency	Tinnitus frequency	8.27 ± 2.8	7.65 ± 3.0	-0.62 ± 2.6
TINSKAL_impairment	Tinnitus impairment	5.16 ± 2.6	4.28 ± 2.6	-0.89 ± 2.3
TINSKAL_loudness	Tinnitus loudness	5.18 ± 2.6	4.67 ± 2.6	-0.50 ± 2.3
TLQ_01_bothears	Tinnitus location: both ears	0.27 ± 0.4	0.28 ± 0.4	0.01 ± 0.4
TLQ_01_entirehead	Tinnitus location: entire head	0.16 ± 0.4	0.15 ± 0.4	-0.01 ± 0.3
TLQ_01_leftear	Tinnitus location: left ear	0.33 ± 0.5	0.33 ± 0.5	0.00 ± 0.3
TLQ_01_rightear	Tinnitus location: right ear	0.25 ± 0.4	0.24 ± 0.4	0.00 ± 0.2
TLQ_02_hissing	Tinnitus noise: hissing	0.10 ± 0.3	0.09 ± 0.3	0.00 ± 0.3
TLQ_02_ringing	Tinnitus noise: ringing	0.03 ± 0.2	0.04 ± 0.2	0.00 ± 0.2
TLQ_02_rustling	Tinnitus noise: rustling	0.28 ± 0.4	0.26 ± 0.4	-0.02 ± 0.3
TLQ_02_whistling	Tinnitus noise: whistling	0.46 ± 0.5	0.49 ± 0.5	0.03 ± 0.4
TQ_auditoryperceptdiff	Auditory perceptual difficulties score	5.12 ± 3.7	4.55 ± 3.5	-0.57 ± 2.1
TQ_cognitivedistress	Cognitive distress score	6.91 ± 4.1	4.94 ± 3.9	-1.98 ± 2.7
TQ_distress	Total tinnitus distress score	38.88 ± 16.8	31.74 ± 17.1	-7.14 ± 9.5
TQ_emodistress	Emotional distress score	10.81 ± 5.5	8.25 ± 5.3	-2.56 ± 3.6
TQ_intrusiveness	Intrusiveness score	10.42 ± 3.6	8.81 ± 3.9	-1.61 ± 2.4
TQ_psychodistress	Psychological distress score	17.72 ± 9.2	13.19 ± 8.9	-4.53 ± 5.7
TQ_sleepdisturbances	Sleep disturbances score	3.46 ± 2.5	3.06 ± 2.5	-0.40 ± 1.3
TQ_somacomplaints	Somatic complaints score	2.16 ± 1.9	2.13 ± 1.9	-0.02 ± 1.2

Table 3: Mean values (± standard deviations) of each phenotype (PT) for each feature at baseline (t_0), after treatment (t_1), and the change with treatment (Δ_{t_0,t_1}).

	PT1 (n=561)			PT2 (n=135)			PT3 (n=157)			PT4 (n=136)		
Feature	$\bar{x}^{t_0}$	$\bar{x}^{t_1}$	$\bar{x}^{\Delta_{t_0,t_1}}$	$\bar{x}^{t_0}$	$\bar{x}^{t_1}$	$\bar{x}^{\Delta_{t_0,t_1}}$	$\bar{x}^{t_0}$	$\bar{x}^{t_1}$	$\bar{x}^{\Delta_{t_0,t_1}}$	$\bar{x}^{t_0}$	$\bar{x}^{t_1}$	$\bar{x}^{\Delta_{t_0,t_1}}$
ACSA_qualityoflife*	4.67 ± 2.3	3.96 ± 2.2	-0.71 ± 2.0	8.23 ± 1.7	7.21 ± 2.2	-1.01 ± 2.0	6.45 ± 2.2	5.47 ± 2.1	-0.98 ± 2.3	7.16 ± 1.9	6.04 ± 2.1	-1.12 ± 1.8
ADSL_depression	10.87 ± 6.5	8.13 ± 7.0	-2.74 ± 6.5	35.54 ± 7.9	27.05 ± 11.4	-8.49 ± 10.1	21.31 ± 5.9	15.61 ± 8.5	-5.70 ± 8.0	26.61 ± 8.0	18.01 ± 9.5	-8.60 ± 9.6
BI_abdominalsymptoms	1.86 ± 2.2	1.45 ± 2.5	-0.41 ± 2.1	6.24 ± 4.5	5.31 ± 4.5	-0.93 ± 3.2	4.75 ± 3.8	3.44 ± 3.5	-1.31 ± 3.2	3.12 ± 3.0	2.54 ± 2.7	-0.58 ± 2.2
BI_fatigue	5.68 ± 4.0	4.86 ± 4.2	-0.82 ± 3.3	16.27 ± 4.0	15.14 ± 5.4	-1.13 ± 4.5	12.50 ± 4.0	10.61 ± 5.2	-1.89 ± 4.1	12.40 ± 4.0	10.75 ± 4.9	-1.65 ± 4.2
BI_heartsymptoms	1.81 ± 2.3	1.54 ± 2.5	-0.27 ± 2.2	7.93 ± 4.6	6.91 ± 4.8	-1.02 ± 4.0	4.37 ± 3.7	3.93 ± 4.1	-0.44 ± 3.2	3.13 ± 2.8	2.65 ± 2.7	-0.48 ± 2.6
BI_limbpain	6.24 ± 4.0	5.67 ± 4.3	-0.57 ± 3.1	14.83 ± 4.5	13.67 ± 5.3	-1.16 ± 3.6	13.18 ± 4.0	11.90 ± 4.7	-1.29 ± 3.7	8.60 ± 4.1	7.93 ± 4.4	-0.66 ± 3.3
BI_overallcomplaints	15.59 ± 9.2	13.52 ± 10.6	-2.07 ± 8.4	45.27 ± 13.3	41.03 ± 16.5	-4.24 ± 12.1	34.80 ± 10.6	29.87 ± 13.6	-4.93 ± 11.5	27.25 ± 9.4	23.88 ± 11.1	-3.37 ± 9.4
BSF_anger	0.49 ± 0.5	0.38 ± 0.5	-0.11 ± 0.5	1.61 ± 0.9	1.26 ± 0.9	-0.35 ± 0.8	0.90 ± 0.6	0.69 ± 0.7	-0.22 ± 0.7	1.23 ± 0.7	0.84 ± 0.7	-0.39 ± 0.7
BSF_anxdepression	0.68 ± 0.5	0.50 ± 0.6	-0.19 ± 0.5	2.62 ± 0.7	2.20 ± 1.0	-0.42 ± 0.8	1.39 ± 0.6	1.02 ± 0.7	-0.37 ± 0.8	1.93 ± 0.6	1.37 ± 0.8	-0.55 ± 0.8
BSF_apathy	0.34 ± 0.4	0.26 ± 0.4	-0.08 ± 0.4	1.59 ± 0.8	1.18 ± 0.9	-0.42 ± 0.8	0.61 ± 0.5	0.41 ± 0.5	-0.19 ± 0.5	1.04 ± 0.7	0.66 ± 0.7	-0.38 ± 0.7
BSF_elevatedmood*	2.15 ± 0.8	1.70 ± 0.9	-0.45 ± 0.7	3.64 ± 0.4	3.18 ± 0.7	-0.46 ± 0.7	3.04 ± 0.6	2.43 ± 0.8	-0.61 ± 0.8	3.32 ± 0.5	2.72 ± 0.8	-0.59 ± 0.7
BSF_fatigue	1.13 ± 0.7	0.83 ± 0.7	-0.30 ± 0.7	2.94 ± 0.7	2.46 ± 1.0	-0.48 ± 0.8	2.08 ± 0.7	1.54 ± 0.9	-0.55 ± 0.9	2.38 ± 0.7	1.75 ± 0.9	-0.64 ± 0.9
BSF_mindset*	1.62 ± 0.7	1.41 ± 0.7	-0.20 ± 0.6	2.61 ± 0.7	2.40 ± 0.7	-0.21 ± 0.6	2.17 ± 0.6	1.92 ± 0.7	-0.25 ± 0.6	2.35 ± 0.6	2.13 ± 0.7	-0.21 ± 0.7
ISR_additionalitems	0.44 ± 0.3	0.39 ± 0.3	-0.06 ± 0.3	1.59 ± 0.5	1.49 ± 0.6	-0.10 ± 0.5	0.87 ± 0.4	0.84 ± 0.5	-0.04 ± 0.4	0.98 ± 0.4	0.89 ± 0.4	-0.08 ± 0.4
ISR_anxiety	0.55 ± 0.6	0.50 ± 0.6	-0.05 ± 0.5	2.12 ± 1.0	2.04 ± 0.9	-0.08 ± 0.9	1.08 ± 0.8	1.07 ± 0.8	-0.01 ± 0.8	1.16 ± 0.8	1.10 ± 0.8	-0.07 ± 0.7
ISR_compulsivesyn	0.46 ± 0.6	0.49 ± 0.6	0.03 ± 0.6	1.71 ± 1.0	1.68 ± 0.9	-0.03 ± 0.9	0.80 ± 0.7	0.89 ± 0.8	0.08 ± 0.8	1.15 ± 0.9	1.10 ± 0.8	-0.05 ± 0.8
ISR_depression	0.63 ± 0.5	0.53 ± 0.6	-0.10 ± 0.5	2.56 ± 0.7	2.36 ± 0.8	-0.20 ± 0.8	1.35 ± 0.6	1.21 ± 0.7	-0.13 ± 0.7	1.90 ± 0.6	1.61 ± 0.7	-0.29 ± 0.7
ISR_eatingdisorder	0.55 ± 0.7	0.51 ± 0.7	-0.03 ± 0.5	0.94 ± 1.0	0.92 ± 1.0	-0.02 ± 0.6	0.84 ± 0.8	0.82 ± 0.9	-0.03 ± 0.7	0.75 ± 0.9	0.68 ± 0.9	-0.07 ± 0.5
ISR_somatosyn	0.31 ± 0.5	0.31 ± 0.5	-0.01 ± 0.4	1.46 ± 1.0	1.36 ± 0.9	-0.09 ± 0.9	0.79 ± 0.8	0.84 ± 0.8	0.06 ± 0.7	0.76 ± 0.8	0.64 ± 0.7	-0.12 ± 0.7
ISR_totalpsychiatricsyn	0.48 ± 0.3	0.44 ± 0.4	-0.04 ± 0.3	1.71 ± 0.5	1.62 ± 0.5	-0.09 ± 0.5	0.94 ± 0.3	0.93 ± 0.5	-0.01 ± 0.5	1.10 ± 0.4	0.99 ± 0.4	-0.11 ± 0.4
PHQK_depression	5.19 ± 3.2	4.11 ± 3.3	-1.08 ± 2.8	16.57 ± 4.5	14.56 ± 5.2	-2.01 ± 4.9	9.91 ± 3.1	8.96 ± 4.0	-0.96 ± 4.2	12.15 ± 3.7	9.78 ± 4.1	-2.38 ± 4.4
PHQK_panicsyn	0.02 ± 0.1	0.03 ± 0.2	0.01 ± 0.2	0.36 ± 0.5	0.35 ± 0.5	-0.01 ± 0.5	0.10 ± 0.3	0.13 ± 0.3	0.03 ± 0.4	0.12 ± 0.3	0.10 ± 0.3	-0.01 ± 0.3
PSQ_demand	0.44 ± 0.2	0.41 ± 0.2	-0.03 ± 0.1	0.64 ± 0.2	0.62 ± 0.2	-0.02 ± 0.1	0.52 ± 0.2	0.51 ± 0.2	-0.01 ± 0.1	0.60 ± 0.2	0.56 ± 0.2	-0.04 ± 0.1
PSQ_joy*	0.41 ± 0.2	0.37 ± 0.2	-0.04 ± 0.1	0.75 ± 0.1	0.76 ± 0.1	0.01 ± 0.1	0.57 ± 0.2	0.56 ± 0.2	-0.02 ± 0.2	0.69 ± 0.1	0.65 ± 0.1	-0.03 ± 0.1
PSQ_stress	0.36 ± 0.1	0.33 ± 0.2	-0.03 ± 0.1	0.69 ± 0.1	0.66 ± 0.1	-0.03 ± 0.1	0.51 ± 0.1	0.48 ± 0.1	-0.03 ± 0.1	0.60 ± 0.1	0.55 ± 0.1	-0.05 ± 0.1
PSQ_tension	0.47 ± 0.2	0.41 ± 0.2	-0.05 ± 0.1	0.84 ± 0.1	0.78 ± 0.2	-0.05 ± 0.1	0.67 ± 0.1	0.60 ± 0.2	-0.07 ± 0.1	0.74 ± 0.2	0.67 ± 0.1	-0.08 ± 0.2
PSQ_worries	0.27 ± 0.2	0.25 ± 0.2	-0.03 ± 0.1	0.69 ± 0.2	0.65 ± 0.2	-0.03 ± 0.2	0.45 ± 0.2	0.40 ± 0.2	-0.04 ± 0.2	0.56 ± 0.2	0.49 ± 0.2	-0.07 ± 0.1
SES_affectivepain	19.24 ± 5.9	18.45 ± 6.1	-0.80 ± 5.3	37.18 ± 10.4	34.60 ± 11.0	-2.58 ± 8.4	31.87 ± 8.7	28.35 ± 9.0	-3.52 ± 10.3	23.34 ± 8.0	21.19 ± 7.4	-2.15 ± 7.5
SES_sensoricpain	11.74 ± 2.6	11.73 ± 3.1	-0.01 ± 2.8	19.81 ± 6.7	19.16 ± 6.6	-0.65 ± 6.0	16.34 ± 5.4	15.88 ± 5.6	-0.46 ± 5.5	12.62 ± 3.2	12.54 ± 3.6	-0.07 ± 3.8
SF8_bodilyhealth*	8.75 ± 9.2	8.28 ± 8.7	-0.47 ± 7.3	22.87 ± 8.8	22.24 ± 9.3	-0.64 ± 7.1	22.37 ± 6.4	20.75 ± 7.1	-1.61 ± 7.9	10.00 ± 8.5	10.55 ± 8.8	0.55 ± 7.8
SF8_mentalcomp*	18.92 ± 8.4	17.76 ± 8.7	-1.16 ± 7.4	40.91 ± 7.5	39.68 ± 8.1	-1.23 ± 7.5	28.36 ± 8.0	27.20 ± 8.7	-1.16 ± 9.3	36.03 ± 7.9	34.35 ± 8.6	-1.68 ± 8.7
SF8_mentalhealth*	9.23 ± 8.1	8.17 ± 8.0	-1.05 ± 7.5	28.35 ± 7.0	27.06 ± 7.9	-1.29 ± 7.8	18.16 ± 8.2	16.26 ± 8.4	-1.91 ± 9.1	23.57 ± 7.7	21.78 ± 8.6	-1.80 ± 9.2
SF8_overallhealth*	14.52 ± 5.8	13.68 ± 5.6	-0.84 ± 5.3	25.89 ± 6.2	24.97 ± 6.5	-0.92 ± 5.5	20.93 ± 4.9	20.10 ± 5.3	-0.83 ± 6.0	18.93 ± 5.7	18.21 ± 5.8	-0.72 ± 5.2
SF8_physicalcomp*	17.15 ± 7.8	17.00 ± 7.4	-0.15 ± 6.2	31.58 ± 8.8	31.18 ± 9.0	-0.41 ± 7.3	29.01 ± 7.5	27.57 ± 7.0	-1.44 ± 7.7	18.01 ± 7.2	18.98 ± 7.8	0.96 ± 7.3
SF8_physicalfunct*	5.13 ± 6.2	5.29 ± 6.3	0.16 ± 6.0	16.91 ± 8.2	17.13 ± 8.2	0.23 ± 7.9	14.30 ± 7.7	12.91 ± 7.7	-1.39 ± 8.1	7.30 ± 6.5	7.43 ± 7.1	0.14 ± 6.3
SF8_roleemotional*	6.00 ± 7.0	5.59 ± 7.3	-0.40 ± 6.6	22.59 ± 7.1	22.24 ± 7.0	-0.35 ± 5.8	14.49 ± 7.9	14.05 ± 7.8	-0.44 ± 8.2	16.02 ± 8.1	15.11 ± 8.3	-0.91 ± 7.1
SF8_rolephysical*	5.10 ± 6.8	4.93 ± 6.5	-0.17 ± 6.2	19.87 ± 8.2	19.09 ± 8.8	-0.78 ± 8.5	14.58 ± 7.7	12.93 ± 7.4	-1.65 ± 8.6	8.92 ± 7.7	9.56 ± 8.0	0.64 ± 9.4
SF8_socialfunct*	4.64 ± 6.3	4.46 ± 6.3	-0.18 ± 6.5	20.92 ± 7.5	20.39 ± 7.7	-0.53 ± 8.4	11.81 ± 7.6	11.29 ± 8.0	-0.52 ± 8.3	14.60 ± 8.1	14.37 ± 7.9	-0.24 ± 8.9
SF8_vitality*	12.75 ± 6.4	11.99 ± 6.8	-0.76 ± 6.1	25.17 ± 6.1	24.69 ± 5.9	-0.48 ± 6.0	19.53 ± 5.7	19.25 ± 5.9	-0.28 ± 6.5	19.79 ± 5.8	20.52 ± 5.9	0.73 ± 6.0
SSKAL_painfrequency	3.49 ± 3.5	3.33 ± 3.4	-0.16 ± 3.3	7.18 ± 2.7	6.81 ± 3.1	-0.38 ± 2.6	6.99 ± 2.8	6.58 ± 2.9	-0.41 ± 3.2	3.91 ± 3.2	3.72 ± 3.1	-0.20 ± 3.2

Continued on next page

Feature	PT1 (n=561)			PT2 (n=135)			PT3 (n=157)			PT4 (n=136)		
	$\bar{x}^{t_0}$	$\bar{x}^{t_1}$	$\bar{x}^{\Delta_{t_0,t_1}}$	$\bar{x}^{t_0}$	$\bar{x}^{t_1}$	$\bar{x}^{\Delta_{t_0,t_1}}$	$\bar{x}^{t_0}$	$\bar{x}^{t_1}$	$\bar{x}^{\Delta_{t_0,t_1}}$	$\bar{x}^{t_0}$	$\bar{x}^{t_1}$	$\bar{x}^{\Delta_{t_0,t_1}}$
SSKAL_painimpairment	2.37 ± 2.2	2.27 ± 2.2	-0.10 ± 2.1	6.78 ± 2.4	6.11 ± 2.7	-0.67 ± 2.4	5.73 ± 2.4	4.96 ± 2.3	-0.77 ± 2.7	3.18 ± 2.6	3.34 ± 2.4	0.16 ± 2.8
SSKAL_painseverity	1.69 ± 1.9	1.81 ± 2.1	0.12 ± 1.8	5.57 ± 2.5	5.39 ± 2.7	-0.18 ± 2.2	4.99 ± 2.2	4.58 ± 2.5	-0.40 ± 2.6	2.24 ± 2.1	2.28 ± 2.1	0.04 ± 2.4
SWOP_optimism*	0.97 ± 0.6	0.89 ± 0.6	-0.09 ± 0.4	2.14 ± 0.7	2.00 ± 0.7	-0.14 ± 0.6	1.30 ± 0.6	1.22 ± 0.7	-0.08 ± 0.6	1.60 ± 0.6	1.58 ± 0.7	-0.02 ± 0.5
SWOP_pessimism	1.88 ± 0.6	1.89 ± 0.7	0.01 ± 0.6	2.72 ± 0.7	2.78 ± 0.6	0.06 ± 0.6	2.24 ± 0.6	2.25 ± 0.7	0.01 ± 0.6	2.31 ± 0.6	2.32 ± 0.7	0.01 ± 0.6
SWOP_selfefficacy*	0.99 ± 0.5	0.92 ± 0.5	-0.07 ± 0.3	1.87 ± 0.5	1.82 ± 0.5	-0.05 ± 0.4	1.32 ± 0.5	1.27 ± 0.6	-0.05 ± 0.4	1.48 ± 0.5	1.39 ± 0.5	-0.09 ± 0.4
TINSKAL_frequency	8.17 ± 2.9	7.38 ± 3.1	-0.78 ± 2.7	8.59 ± 2.7	8.26 ± 2.9	-0.33 ± 2.7	8.40 ± 2.8	8.16 ± 2.6	-0.24 ± 2.6	8.26 ± 2.9	7.59 ± 3.0	-0.67 ± 2.1
TINSKAL_impairment	4.27 ± 2.2	3.36 ± 2.2	-0.91 ± 2.0	6.84 ± 2.7	6.41 ± 2.6	-0.43 ± 2.9	6.11 ± 2.4	5.27 ± 2.3	-0.84 ± 2.5	6.07 ± 2.4	4.78 ± 2.5	-1.29 ± 2.2
TINSKAL_loudness	4.49 ± 2.4	3.90 ± 2.3	-0.59 ± 2.1	6.42 ± 2.6	6.20 ± 2.6	-0.22 ± 2.6	5.88 ± 2.4	5.62 ± 2.5	-0.26 ± 2.3	5.97 ± 2.6	5.25 ± 2.7	-0.71 ± 2.3
TLQ_01_bothears	0.26 ± 0.4	0.26 ± 0.4	0.00 ± 0.4	0.30 ± 0.5	0.32 ± 0.5	0.02 ± 0.5	0.29 ± 0.5	0.29 ± 0.5	0.00 ± 0.4	0.24 ± 0.4	0.27 ± 0.4	0.04 ± 0.3
TLQ_01_entirehead	0.12 ± 0.3	0.12 ± 0.3	0.00 ± 0.3	0.27 ± 0.4	0.24 ± 0.4	-0.03 ± 0.4	0.16 ± 0.4	0.16 ± 0.4	0.00 ± 0.3	0.18 ± 0.4	0.18 ± 0.4	0.00 ± 0.3
TLQ_01_leftear	0.35 ± 0.5	0.36 ± 0.5	0.01 ± 0.3	0.25 ± 0.4	0.26 ± 0.4	0.01 ± 0.3	0.29 ± 0.5	0.28 ± 0.5	-0.01 ± 0.3	0.33 ± 0.5	0.32 ± 0.5	-0.01 ± 0.2
TLQ_01_rightear	0.26 ± 0.4	0.25 ± 0.4	-0.01 ± 0.2	0.18 ± 0.4	0.19 ± 0.4	0.01 ± 0.2	0.25 ± 0.4	0.26 ± 0.4	0.01 ± 0.2	0.25 ± 0.4	0.23 ± 0.4	-0.02 ± 0.2
TLQ_02_hissing	0.10 ± 0.3	0.10 ± 0.3	0.00 ± 0.3	0.05 ± 0.2	0.06 ± 0.2	0.01 ± 0.3	0.11 ± 0.3	0.08 ± 0.3	-0.03 ± 0.3	0.11 ± 0.3	0.10 ± 0.3	-0.01 ± 0.2
TLQ_02_ringing	0.03 ± 0.2	0.03 ± 0.2	0.00 ± 0.2	0.02 ± 0.1	0.07 ± 0.3	0.05 ± 0.2	0.04 ± 0.2	0.04 ± 0.2	0.00 ± 0.1	0.07 ± 0.2	0.02 ± 0.1	-0.04 ± 0.2
TLQ_02_rustling	0.29 ± 0.5	0.27 ± 0.4	-0.03 ± 0.3	0.24 ± 0.4	0.25 ± 0.4	0.01 ± 0.3	0.31 ± 0.5	0.29 ± 0.5	-0.02 ± 0.4	0.21 ± 0.4	0.18 ± 0.4	-0.03 ± 0.2
TLQ_02_whistling	0.46 ± 0.5	0.48 ± 0.5	0.02 ± 0.4	0.44 ± 0.5	0.46 ± 0.5	0.02 ± 0.4	0.41 ± 0.5	0.45 ± 0.5	0.04 ± 0.4	0.50 ± 0.5	0.59 ± 0.5	0.09 ± 0.4
TQ_auditoryperceptdiff	3.80 ± 3.1	3.32 ± 3.0	-0.48 ± 2.0	7.96 ± 3.4	7.44 ± 3.5	-0.53 ± 2.3	6.76 ± 3.5	6.05 ± 3.5	-0.71 ± 2.2	5.86 ± 3.6	5.00 ± 3.5	-0.86 ± 1.9
TQ_cognitivedistress	5.06 ± 3.3	3.33 ± 2.9	-1.73 ± 2.4	11.08 ± 3.1	8.88 ± 4.0	-2.20 ± 2.8	8.48 ± 3.5	6.26 ± 3.6	-2.22 ± 2.9	8.62 ± 3.8	6.15 ± 3.9	-2.48 ± 3.4
TQ_distress	29.42 ± 12.6	23.09 ± 12.1	-6.32 ± 8.8	58.60 ± 12.3	51.18 ± 15.8	-7.42 ± 9.6	49.51 ± 12.7	40.97 ± 14.9	-8.54 ± 10.5	46.09 ± 11.9	37.49 ± 14.2	-8.60 ± 10.3
TQ_emodistress	7.80 ± 4.2	5.64 ± 3.8	-2.16 ± 3.4	17.06 ± 4.2	14.33 ± 4.8	-2.73 ± 3.7	13.58 ± 4.3	10.43 ± 4.7	-3.15 ± 4.2	13.82 ± 4.1	10.49 ± 4.8	-3.32 ± 3.6
TQ_intrusiveness	8.84 ± 3.4	7.18 ± 3.4	-1.66 ± 2.5	13.47 ± 2.4	12.09 ± 3.0	-1.38 ± 2.0	12.31 ± 2.5	10.56 ± 3.1	-1.75 ± 2.4	11.74 ± 3.1	10.24 ± 3.8	-1.50 ± 2.6
TQ_psychodistress	12.86 ± 7.0	8.96 ± 6.3	-3.89 ± 5.1	28.14 ± 6.8	23.21 ± 8.5	-4.93 ± 6.0	22.06 ± 7.3	16.69 ± 7.8	-5.36 ± 6.5	22.44 ± 7.3	16.64 ± 8.2	-5.80 ± 6.4
TQ_sleepdisturbances	2.53 ± 2.3	2.21 ± 2.3	-0.32 ± 1.2	5.16 ± 2.2	4.80 ± 2.3	-0.36 ± 1.4	4.84 ± 2.4	4.30 ± 2.5	-0.54 ± 1.4	4.01 ± 2.3	3.42 ± 2.3	-0.60 ± 1.5
TQ_somacomplaints	1.39 ± 1.5	1.41 ± 1.5	0.02 ± 1.1	3.87 ± 1.8	3.64 ± 1.9	-0.22 ± 1.3	3.54 ± 1.9	3.36 ± 2.0	-0.18 ± 1.4	2.04 ± 1.5	2.20 ± 1.6	0.16 ± 1.3

Supplementary C: changes with treatment for each pathway of change

Tables 4 depict the mean values for each feature at baseline (t_0), after treatment (t_1), and the change with treatment (Δ_{t_0,t_1}) for each pathway of change. Higher values for $\bar{x}^{t_0}$ and $\bar{x}^{t_1}$ represent higher symptom burden. Positive values for $\bar{x}^{\Delta_{t_0,t_1}}$ describe an increase in symptom burden.

Table 4: Mean values (± standard deviations) of each pathway of change (PC) for each feature at baseline (t_0), after treatment (t_1), and the change with treatment (Δ_{t_0,t_1}).

	PC1 (n=45)			PC2 (n=217)			PC3 (n=341)			PC4 (n=289)			PC5 (n=97)		
Feature	$\bar{x}^{t_0}$	$\bar{x}^{t_1}$	$\bar{x}^{\Delta_{t_0,t_1}}$	$\bar{x}^{t_0}$	$\bar{x}^{t_1}$	$\bar{x}^{\Delta_{t_0,t_1}}$	$\bar{x}^{t_0}$	$\bar{x}^{t_1}$	$\bar{x}^{\Delta_{t_0,t_1}}$	$\bar{x}^{t_0}$	$\bar{x}^{t_1}$	$\bar{x}^{\Delta_{t_0,t_1}}$			
ACSA_qualityoflife*	6.45 ± 2.7	7.28 ± 2.0	0.83 ± 2.7	5.81 ± 2.6	5.51 ± 2.3	-0.29 ± 1.8	5.20 ± 2.6	4.57 ± 2.5	-0.63 ± 1.7	5.95 ± 2.5	4.61 ± 2.4	-1.34 ± 2.1	6.95 ± 2.3	4.77 ± 2.3	-2.18 ± 2.1
ADSL_depression	24.89 ± 12.5	28.93 ± 13.4	4.04 ± 8.6	17.48 ± 11.3	17.31 ± 10.6	-0.18 ± 6.9	15.53 ± 11.5	12.07 ± 10.3	-3.46 ± 5.8	18.22 ± 10.2	10.58 ± 8.6	-7.64 ± 6.9	24.59 ± 9.9	9.08 ± 6.6	-15.51 ± 7.5
BI_abdominalsymptoms	4.02 ± 3.6	6.47 ± 5.3	2.44 ± 5.0	3.27 ± 3.5	3.17 ± 3.6	-0.10 ± 2.0	2.47 ± 3.2	2.00 ± 3.0	-0.46 ± 1.9	3.42 ± 3.5	2.10 ± 2.9	-1.32 ± 2.4	3.46 ± 3.4	1.52 ± 2.5	-1.95 ± 2.6
BI_fatigue	10.62 ± 6.1	15.29 ± 6.3	4.67 ± 4.8	9.24 ± 5.7	10.27 ± 5.7	1.03 ± 2.7	7.76 ± 5.8	7.04 ± 5.9	-0.72 ± 2.4	9.51 ± 5.6	7.10 ± 5.5	-2.42 ± 2.8	11.93 ± 4.4	5.45 ± 4.3	-6.47 ± 3.8
BI_heartsymptoms	4.96 ± 4.8	8.56 ± 5.9	3.60 ± 5.3	3.33 ± 3.6	3.65 ± 4.0	0.32 ± 2.1	2.62 ± 3.4	2.23 ± 3.3	-0.39 ± 1.9	3.11 ± 3.3	2.37 ± 3.0	-0.74 ± 2.1	4.74 ± 4.6	1.59 ± 2.2	-3.15 ± 3.3
BI_limbpain	11.18 ± 5.1	14.62 ± 4.8	3.44 ± 4.0	8.94 ± 5.4	9.83 ± 5.7	0.89 ± 2.6	7.71 ± 5.4	7.39 ± 5.5	-0.32 ± 2.2	9.13 ± 5.1	7.22 ± 4.9	-1.91 ± 2.7	10.60 ± 4.7	5.90 ± 3.6	-4.70 ± 4.0
BI_overallcomplaints	30.78 ± 16.4	44.93 ± 18.5	14.16 ± 15.9	24.78 ± 15.0	26.93 ± 15.6	2.14 ± 6.1	20.55 ± 15.0	18.67 ± 15.0	-1.89 ± 5.6	25.17 ± 14.1	18.79 ± 13.3	-6.38 ± 6.9	30.73 ± 13.0	14.45 ± 9.7	-16.28 ± 10.0
BSF_anger	1.00 ± 0.9	1.56 ± 1.0	0.56 ± 0.9	0.84 ± 0.8	0.87 ± 0.8	0.02 ± 0.5	0.66 ± 0.7	0.53 ± 0.6	-0.13 ± 0.4	0.84 ± 0.7	0.48 ± 0.5	-0.36 ± 0.5	1.10 ± 0.8	0.29 ± 0.4	-0.81 ± 0.7
BSF_anxdepression	1.72 ± 1.0	2.35 ± 1.1	0.64 ± 0.9	1.19 ± 0.9	1.23 ± 1.0	0.05 ± 0.5	1.04 ± 0.9	0.84 ± 0.9	-0.20 ± 0.4	1.23 ± 0.9	0.71 ± 0.7	-0.52 ± 0.5	1.77 ± 0.9	0.57 ± 0.5	-1.20 ± 0.7
BSF_apathy	0.91 ± 0.9	1.45 ± 1.2	0.54 ± 0.9	0.71 ± 0.7	0.65 ± 0.7	-0.06 ± 0.5	0.52 ± 0.7	0.43 ± 0.7	-0.10 ± 0.4	0.61 ± 0.6	0.28 ± 0.4	-0.33 ± 0.5	0.97 ± 0.8	0.25 ± 0.4	-0.72 ± 0.7
BSF_elevatedmood*	2.94 ± 1.0	3.06 ± 1.0	0.12 ± 0.7	2.61 ± 1.0	2.52 ± 1.0	-0.09 ± 0.5	2.46 ± 1.0	2.11 ± 1.0	-0.35 ± 0.5	2.71 ± 0.8	1.94 ± 0.9	-0.77 ± 0.6	3.18 ± 0.7	1.76 ± 0.9	-1.42 ± 0.9
BSF_fatigue	2.05 ± 0.9	2.49 ± 1.0	0.44 ± 0.7	1.72 ± 1.0	1.67 ± 1.0	-0.05 ± 0.6	1.48 ± 1.0	1.22 ± 1.0	-0.26 ± 0.6	1.73 ± 0.9	1.07 ± 0.8	-0.67 ± 0.7	2.19 ± 0.8	0.77 ± 0.6	-1.42 ± 0.7
BSF_mindset*	2.14 ± 0.8	2.45 ± 0.8	0.31 ± 0.8	1.93 ± 0.7	1.94 ± 0.8	0.01 ± 0.5	1.81 ± 0.8	1.67 ± 0.8	-0.14 ± 0.5	1.95 ± 0.7	1.61 ± 0.7	-0.34 ± 0.6	2.31 ± 0.7	1.47 ± 0.8	-0.84 ± 0.9
ISR_additionalitems	0.77 ± 0.7	1.50 ± 0.9	0.73 ± 0.8	0.76 ± 0.6	0.80 ± 0.6	0.03 ± 0.2	0.66 ± 0.5	0.61 ± 0.5	-0.05 ± 0.2	0.73 ± 0.5	0.57 ± 0.4	-0.16 ± 0.2	1.01 ± 0.5	0.61 ± 0.4	-0.41 ± 0.3
ISR_anxiety	1.00 ± 1.0	2.01 ± 1.2	1.01 ± 1.1	0.99 ± 0.9	1.09 ± 1.0	0.10 ± 0.5	0.77 ± 0.8	0.74 ± 0.8	-0.04 ± 0.5	0.92 ± 0.9	0.77 ± 0.8	-0.15 ± 0.5	1.37 ± 0.9	0.74 ± 0.8	-0.63 ± 0.7
ISR_compulsivesyn	0.69 ± 0.8	1.62 ± 1.2	0.93 ± 1.1	0.73 ± 0.9	0.89 ± 0.8	0.16 ± 0.6	0.67 ± 0.8	0.69 ± 0.8	0.02 ± 0.6	0.83 ± 0.9	0.74 ± 0.8	-0.09 ± 0.6	1.17 ± 1.0	0.74 ± 0.8	-0.43 ± 0.8
ISR_depression	1.28 ± 1.1	2.21 ± 1.1	0.93 ± 1.0	1.16 ± 0.9	1.30 ± 1.0	0.14 ± 0.5	0.99 ± 0.9	0.90 ± 0.9	-0.09 ± 0.4	1.20 ± 0.8	0.86 ± 0.8	-0.34 ± 0.5	1.77 ± 0.8	0.85 ± 0.7	-0.92 ± 0.6
ISR_eatingdisorder	0.55 ± 0.9	0.92 ± 1.2	0.37 ± 0.9	0.66 ± 0.8	0.66 ± 0.8	0.00 ± 0.4	0.61 ± 0.7	0.61 ± 0.8	0.00 ± 0.4	0.78 ± 0.8	0.67 ± 0.8	-0.11 ± 0.6	0.70 ± 0.9	0.48 ± 0.8	-0.22 ± 0.6
ISR_somatosyn	0.47 ± 0.6	1.22 ± 1.2	0.76 ± 1.1	0.69 ± 0.9	0.76 ± 0.9	0.07 ± 0.6	0.47 ± 0.7	0.48 ± 0.7	0.00 ± 0.4	0.60 ± 0.8	0.48 ± 0.7	-0.12 ± 0.5	0.95 ± 1.0	0.54 ± 0.7	-0.41 ± 0.6
ISR_totalpsychiatricsyn	0.79 ± 0.6	1.57 ± 0.8	0.78 ± 0.8	0.82 ± 0.6	0.90 ± 0.6	0.08 ± 0.2	0.69 ± 0.6	0.66 ± 0.6	-0.03 ± 0.2	0.83 ± 0.5	0.67 ± 0.5	-0.16 ± 0.2	1.14 ± 0.5	0.65 ± 0.4	-0.49 ± 0.3
PHQK_depression	8.62 ± 6.3	14.89 ± 6.7	6.27 ± 6.4	8.44 ± 5.3	8.60 ± 5.6	0.16 ± 2.5	7.33 ± 5.6	6.21 ± 5.2	-1.12 ± 2.2	8.63 ± 4.9	6.18 ± 4.3	-2.45 ± 2.6	11.78 ± 4.5	5.86 ± 3.5	-5.93 ± 3.5
PHQK_panicsyn	0.09 ± 0.3	0.38 ± 0.5	0.29 ± 0.5	0.12 ± 0.3	0.12 ± 0.3	0.00 ± 0.3	0.06 ± 0.2	0.09 ± 0.3	0.03 ± 0.2	0.09 ± 0.3	0.05 ± 0.2	-0.04 ± 0.3	0.09 ± 0.3	0.07 ± 0.3	-0.02 ± 0.3
PSQ_demand	0.54 ± 0.2	0.62 ± 0.2	0.08 ± 0.2	0.50 ± 0.2	0.53 ± 0.2	0.03 ± 0.1	0.46 ± 0.2	0.43 ± 0.2	-0.02 ± 0.1	0.52 ± 0.2	0.45 ± 0.2	-0.07 ± 0.1	0.62 ± 0.2	0.48 ± 0.2	-0.14 ± 0.1
PSQ_joy*	0.60 ± 0.2	0.72 ± 0.2	0.12 ± 0.2	0.50 ± 0.2	0.54 ± 0.2	0.05 ± 0.1	0.48 ± 0.2	0.45 ± 0.2	-0.03 ± 0.1	0.53 ± 0.2	0.47 ± 0.2	-0.06 ± 0.1	0.63 ± 0.2	0.49 ± 0.2	-0.14 ± 0.2
PSQ_stress	0.52 ± 0.1	0.63 ± 0.2	0.11 ± 0.1	0.45 ± 0.2	0.49 ± 0.2	0.04 ± 0.1	0.42 ± 0.2	0.39 ± 0.2	-0.03 ± 0.1	0.48 ± 0.2	0.41 ± 0.2	-0.07 ± 0.1	0.58 ± 0.2	0.43 ± 0.2	-0.16 ± 0.1
PSQ_tension	0.64 ± 0.2	0.75 ± 0.2	0.11 ± 0.2	0.56 ± 0.2	0.59 ± 0.2	0.03 ± 0.1	0.54 ± 0.2	0.49 ± 0.2	-0.05 ± 0.1	0.61 ± 0.2	0.51 ± 0.2	-0.11 ± 0.1	0.73 ± 0.2	0.50 ± 0.2	-0.23 ± 0.2
PSQ_worries	0.47 ± 0.2	0.61 ± 0.2	0.13 ± 0.2	0.39 ± 0.2	0.43 ± 0.2	0.04 ± 0.1	0.35 ± 0.2	0.32 ± 0.2	-0.03 ± 0.1	0.40 ± 0.2	0.32 ± 0.2	-0.09 ± 0.1	0.51 ± 0.2	0.35 ± 0.2	-0.16 ± 0.1
SES_affectivepain	26.22 ± 11.6	35.62 ± 12.4	9.40 ± 12.2	24.01 ± 10.2	24.43 ± 10.4	0.42 ± 5.3	22.43 ± 9.4	21.67 ± 9.0	-0.76 ± 4.7	24.82 ± 9.6	21.28 ± 8.1	-3.54 ± 6.1	28.68 ± 11.4	19.65 ± 7.4	-9.03 ± 8.2
SES_sensoricpain	15.31 ± 6.3	19.44 ± 7.3	4.13 ± 8.1	13.47 ± 4.7	14.42 ± 5.7	0.96 ± 3.5	13.13 ± 4.7	13.12 ± 4.7	-0.01 ± 3.0	13.72 ± 4.7	12.84 ± 4.0	-0.88 ± 3.3	15.34 ± 5.6	12.12 ± 3.4	-3.22 ± 4.7
SF8_bodilyhealth*	19.04 ± 10.3	21.37 ± 10.2	2.33 ± 9.4	13.27 ± 10.5	14.39 ± 10.6	1.12 ± 6.1	10.90 ± 10.4	11.20 ± 10.3	0.31 ± 7.3	13.85 ± 10.7	11.73 ± 9.7	-2.12 ± 7.2	14.59 ± 10.4	10.80 ± 9.7	-3.79 ± 8.4
SF8_mentalcomp*	29.56 ± 12.8	36.37 ± 10.2	6.81 ± 8.9	23.52 ± 11.7	27.72 ± 12.3	4.20 ± 6.8	23.89 ± 12.2	21.92 ± 12.2	-1.98 ± 5.9	27.02 ± 10.9	22.89 ± 10.8	-4.13 ± 7.6	31.97 ± 9.8	26.00 ± 10.7	-5.97 ± 8.1
SF8_mentalhealth*	18.70 ± 11.2	24.37 ± 9.5	5.67 ± 8.7	13.22 ± 10.4	16.62 ± 11.2	3.40 ± 7.2	13.67 ± 11.3	11.89 ± 11.1	-1.77 ± 6.3	16.25 ± 10.1	12.35 ± 9.5	-3.90 ± 7.9	20.57 ± 9.9	14.67 ± 9.6	-5.89 ± 8.9
SF8_overallhealth*	20.96 ± 7.3	24.08 ± 6.5	3.12 ± 7.8	17.23 ± 6.9	18.20 ± 7.2	0.97 ± 4.5	16.75 ± 7.5	15.83 ± 7.1	-0.92 ± 4.9	17.80 ± 6.3	15.79 ± 6.3	-2.01 ± 5.3	20.25 ± 6.2	17.37 ± 6.2	-2.89 ± 6.1
SF8_physicalcomp*	26.42 ± 10.1	30.61 ± 8.7	4.18 ± 9.5	21.59 ± 9.4	22.82 ± 9.5	1.22 ± 6.3	19.36 ± 9.3	19.37 ± 9.3	0.01 ± 5.9	21.57 ± 10.0	20.10 ± 8.9	-1.47 ± 6.5	22.44 ± 10.2	19.67 ± 8.5	-2.77 ± 8.0
SF8_physicalfunct*	12.27 ± 9.0	15.53 ± 9.3	3.25 ± 9.6	8.94 ± 7.9	10.00 ± 8.1	1.05 ± 6.3	7.09 ± 8.1	7.28 ± 8.2	0.18 ± 6.2	8.70 ± 8.2	7.78 ± 7.6	-0.93 ± 6.1	9.99 ± 8.6	7.44 ± 8.1	-2.55 ± 8.3
SF8_roleemotional*	14.02 ± 10.2	19.34 ± 8.3	5.31 ± 7.7	9.96 ± 9.7	12.93 ± 9.8	2.98 ± 6.6	9.38 ± 9.5	8.17 ± 9.1	-1.21 ± 5.8	12.00 ± 9.2	9.62 ± 9.1	-2.39 ± 6.4	14.51 ± 8.9	11.96 ± 9.5	-2.55 ± 7.3
SF8_rolephysical*	13.17 ± 9.9	18.65 ± 8.0	5.48 ± 10.8	8.88 ± 8.8	10.83 ± 8.9	1.95 ± 6.8	7.79 ± 8.8	7.08 ± 8.5	-0.70 ± 6.3	9.71 ± 8.9	7.86 ± 8.2	-1.85 ± 7.3	11.01 ± 9.7	8.24 ± 8.0	-2.77 ± 8.8
SF8_socialfunct*	12.99 ± 10.5	17.83 ± 8.0	4.84 ± 10.1	8.45 ± 8.9	11.38 ± 9.5	2.93 ± 7.4	7.78 ± 9.2	7.49 ± 9.1	-0.29 ± 5.9	10.02 ± 8.7	7.82 ± 8.2	-2.20 ± 6.9	13.43 ± 8.6	9.25 ± 8.3	-4.19 ± 8.1
SF8_vitality*	19.66 ± 9.0	24.28 ± 6.2	4.62 ± 7.1	15.88 ± 7.8	17.96 ± 7.8	2.08 ± 5.5	15.21 ± 8.0	14.35 ± 8.3	-0.87 ± 5.5	17.04 ± 7.1	15.36 ± 7.4	-1.68 ± 5.9	19.23 ± 6.3	16.02 ± 7.1	-3.21 ± 7.1
SSKAL_painfrequency	5.49 ± 3.9	7.02 ± 3.2	1.53 ± 4.5	4.76 ± 3.7	4.72 ± 3.6	-0.04 ± 3.1	4.03 ± 3.6	4.38 ± 3.7	0.36 ± 2.9	4.76 ± 3.6	3.95 ± 3.4	-0.82 ± 3.0	5.46 ± 3.5	3.63 ± 3.4	-1.83 ± 3.0
SSKAL_painimpairment	4.62 ± 3.3	6.35 ± 2.9	1.73 ± 4.2	3.68 ± 2.9	3.68 ± 2.8	0.00 ± 2.2	3.04 ± 2.7	3.19 ± 2.7	0.15 ± 2.0	3.62 ± 2.8	3.00 ± 2.5	-0.62 ± 1.9	5.03 ± 2.9	3.00 ± 2.2	-2.03 ± 2.6
SSKAL_painseverity	3.57 ± 3.1	5.63 ± 3.0	2.07 ± 3.7	2.94 ± 2.6	3.10 ± 2.7	0.17 ± 2.0	2.34 ± 2.4	2.66 ± 2.6	0.33 ± 1.7	2.91 ± 2.6	2.56 ± 2.4	-0.34 ± 1.7	3.62 ± 2.6	2.02 ± 2.0	-1.60 ± 2.0
SWOP_optimism*	1.32 ± 0.8	1.73 ± 0.9	0.41 ± 0.7	1.28 ± 0.8	1.36 ± 0.8	0.08 ± 0.5	1.21 ± 0.8	1.12 ± 0.8	-0.08 ± 0.4	1.21 ± 0.7	1.04 ± 0.7	-0.17 ± 0.5	1.63 ± 0.8	1.21 ± 0.8	-0.43 ± 0.6
SWOP_pessimism	2.18 ± 0.7	2.61 ± 0.8	0.43 ± 0.7	2.15 ± 0.7	2.28 ± 0.7	0.13 ± 0.6	2.06 ± 0.7	2.06 ± 0.7	0.01 ± 0.6	2.07 ± 0.7	2.03 ± 0.7	-0.04 ± 0.6	2.29 ± 0.7	2.07 ± 0.8	-0.23 ± 0.5
SWOP_selfefficacy*	1.45 ± 0.7	1.63 ± 0.7	0.18 ± 0.5	1.25 ± 0.6	1.32 ± 0.6	0.07 ± 0.3	1.16 ± 0.6	1.07 ± 0.5	-0.09 ± 0.3	1.20 ± 0.5	1.11 ± 0.5	-0.09 ± 0.4	1.42 ± 0.6	1.08 ± 0.6	-0.34 ± 0.4
TINSKAL_frequency	8.85 ± 2.5	8.13 ± 2.8	-0.72 ± 3.3	8.09 ± 3.1	7.66 ± 3.1	-0.43 ± 2.7	8.03 ± 2.9	7.89 ± 2.9	-0.14 ± 2.3	8.45 ± 2.7	7.42 ± 3.1	-1.03 ± 2.5	8.77 ± 2.4	7.31 ± 3.1	-1.45 ± 2.9
TINSKAL_impairment	6.27 ± 2.6	6.19 ± 2.8	-0.09 ± 2.8	5.19 ± 2.7	4.61 ± 2.7	-0.58 ± 1.9	4.51 ± 2.5	4.11 ± 2.5	-0.40 ± 2.2	5.31 ± 2.4	3.96 ± 2.5	-1.35 ± 2.1	6.43 ± 2.2	4.14 ± 2.2	-2.29 ± 2.7
TINSKAL_loudness	6.20 ± 2.8	6.27 ± 3.0	0.07 ± 3.0	5.13 ± 2.6	4.78 ± 2.7	-0.35 ± 2.3	4.73 ± 2.5	4.50 ± 2.6	-0.23 ± 2.1	5.27 ± 2.5	4.60 ± 2.5	-0.67 ± 2.3	6.10 ± 2.4	4.49 ± 2.5	-1.62 ± 2.1
TLQ_01_bothears	0.31 ± 0.5	0.40 ± 0.5	0.09 ± 0.5	0.26 ± 0.4	0.27 ± 0.4	0.01 ± 0.4	0.29 ± 0.5	0.28 ± 0.4	-0.01 ± 0.3	0.24 ± 0.4	0.25 ± 0.4	0.01 ± 0.4	0.29 ± 0.5	0.31 ± 0.5	0.02 ± 0.4
TLQ_01_entirehead	0.13 ± 0.3	0.09 ± 0.3	-0.04 ± 0.4	0.14 ± 0.3	0.13 ± 0.3	0.00 ± 0.3	0.15 ± 0.4	0.14 ± 0.3	-0.01 ± 0.3	0.19 ± 0.4	0.20 ± 0.4	0.01 ± 0.3	0.14 ± 0.4	0.11 ± 0.3	-0.03 ± 0.3
TLQ_01_leftear	0.38 ± 0.5	0.29 ± 0.5	-0.09 ± 0.4	0.36 ± 0.5	0.36 ± 0.5	0.00 ± 0.3	0.32 ± 0.5	0.33 ± 0.5	0.02 ± 0.2	0.32 ± 0.5	0.31 ± 0.5	-0.01 ± 0.3	0.27 ± 0.4	0.29 ± 0.5	0.02 ± 0.2
TLQ_01_rightear	0.18 ± 0.4	0.20 ± 0.4	0.02 ± 0.3	0.24 ± 0.4	0.23 ± 0.4	0.00 ± 0.2	0.24 ± 0.4	0.25 ± 0.4	0.00 ± 0.2	0.25 ± 0.4	0.24 ± 0.4	-0.01 ± 0.2	0.30 ± 0.5	0.28 ± 0.5	-0.02 ± 0.2
TLQ_02_hissing	0.02 ± 0.1	0.07 ± 0.3	0.04 ± 0.2	0.10 ± 0.3	0.08 ± 0.3	-0.02 ± 0.2	0.12 ± 0.3	0.11 ± 0.3	-0.01 ± 0.3	0.09 ± 0.3	0.10 ± 0.3	0.01 ± 0.3	0.05 ± 0.2	0.07 ± 0.3	0.02 ± 0.2
TLQ_02_ringing	0.02 ± 0.1	0.02 ± 0.1	0.00 ± 0.2	0.01 ± 0.1	0.04 ± 0.2	0.03 ± 0.2	0.05 ± 0.2	0.04 ± 0.2	-0.01 ± 0.2	0.03 ± 0.2	0.03 ± 0.2	0.00 ± 0.1	0.05 ± 0.2	0.05 ± 0.2	0.00 ± 0.2
TLQ_02_rustling	0.24 ± 0.4	0.16 ± 0.4	-0.09 ± 0.4	0.30 ± 0.5	0.28 ± 0.4	-0.03 ± 0.3	0.27 ± 0.4	0.23 ± 0.4	-0.04 ± 0.3	0.29 ± 0.5	0.29 ± 0.5	0.00 ± 0.3	0.25 ± 0.4	0.25 ± 0.4	0.00 ± 0.3
TLQ_02_whistling	0.56 ± 0.5	0.62 ± 0.5	0.07 ± 0.5	0.45 ± 0.5	0.47 ± 0.5	0.01 ± 0.3	0.45 ± 0.5	0.51 ± 0.5	0.06 ± 0.4	0.45 ± 0.5	0.45 ± 0.5	0.00 ± 0.4	0.48 ± 0.5	0.52 ± 0.5	0.03 ± 0.4
TQ_auditoryperceptdiff	7.24 ± 4.0	8.51 ± 3.7	1.27 ± 3.2	5.25 ± 3.6	5.48 ± 3.5	0.23 ± 1.9	4.27 ± 3.5	3.97 ± 3.4	-0.30 ± 1.6	5.45 ± 3.6	4.15 ± 3.4	-1.30 ± 1.9	5.84 ± 3.5	3.85 ± 3.0	-1.99 ± 2.3
TQ_cognitivedistress	7.84 ± 4.3	8.91 ± 4.6	1.07 ± 2.8	6.90 ± 4.1	6.12 ± 4.1	-0.78 ± 2.1	5.88 ± 4.1	4.64 ± 3.9	-1.24 ± 2.0	7.23 ± 3.7	4.13 ± 3.3	-3.10 ± 2.2	9.21 ± 3.7	3.93 ± 3.0	-5.28 ± 2.8
TQ_distress	48.07 ± 18.3	52.80 ± 17.5	4.73 ± 12.7	38.31 ± 17.1	37.64 ± 17.0	-0.67 ± 6.8	33.89 ± 17.2	29.16 ± 17.0	-4.73 ± 5.4	40.78 ± 15.0	28.49 ± 14.9	-12.29 ± 6.9	47.78 ± 12.9	27.55 ± 12.2	-20.24 ± 8.1
TQ_emodistress	13.11 ± 6.1	14.53 ± 6.0	1.42 ± 5.0	10.41 ± 5.4	9.98 ± 5.4	-0.43 ± 2.8	9.22 ± 5.6	7.50 ± 5.3	-1.72 ± 2.4	11.54 ± 5.0	7.28 ± 4.6	-4.26 ± 2.9	14.04 ± 4.8	7.01 ± 3.9	-7.03 ± 3.4
TQ_intrusiveness	12.22 ± 3.7	12.47 ± 3.2	0.24 ± 2.5	10.27 ± 3.8	9.90 ± 3.8	-0.37 ± 2.1	9.55 ± 3.8	8.37 ± 3.9	-1.18 ± 1.9	10.82 ± 3.3	8.23 ± 3.6	-2.59 ± 2.3	11.80 ± 3.0	7.94 ± 3.5	-3.87 ± 2.4
TQ_psychodistress	20.96 ± 10.2	23.44 ± 10.3	2.49 ± 7.3	17.30 ± 9.2	16.10 ± 9.2	-1.21 ± 4.2	15.10 ± 9.2	12.14 ± 8.8	-2.96 ± 3.5	18.77 ± 8.2	11.41 ± 7.5	-7.36 ± 4.3	23.25 ± 8.0	10.94 ± 6.2	-12.31 ± 5.4
TQ_sleepdisturbances	4.40 ± 2.7	4.60 ± 2.4	0.20 ± 1.4	3.54 ± 2.7	3.60 ± 2.6	0.06 ± 1.2	3.09 ± 2.5	2.80 ± 2.5	-0.28 ± 1.1	3.42 ± 2.4	2.83 ± 2.5	-0.59 ± 1.2	4.29 ± 2.3	2.75 ± 2.3	-1.54 ± 1.7
TQ_somacomplaints	3.24 ± 2.3	3.78 ± 2.1	0.53 ± 1.5	1.94 ± 1.9	2.56 ± 1.9	0.62 ± 1.1	1.89 ± 1.9	1.88 ± 1.9	-0.01 ± 1.1	2.32 ± 1.8	1.88 ± 1.8	-0.44 ± 1.2	2.61 ± 1.9	2.07 ± 1.7	-0.54 ± 1.2

Supplementary D: comparison of dimensionality reduction methods

Dimensionality reduction (DR) is the transformation of data from a high-dimensional space to a low-dimensional space with the goal of preserving the most important properties of the original data, such as pairwise distances, clusters, and outliers. Projecting to a two- or three-dimensional space allows common visualization types such as scatterplots to be used. While linear DR techniques like principal component analysis[3] (PCA) are still commonly used, newer techniques like t-stochastic neighborhood embedding[4] (t-SNE) and uniform manifold approximation and projection[5] (UMAP) have been shown to better capture nonlinear properties of the original data. We use UMAP because it is widely adopted in communities dealing with high-dimensional data like population genetics,[6] e.g. to discover phenotype heterogeneity in large genomic cohort data[7] and to support multiomics analysis by highlighting epigenetic heterogeneity within and common malignant cell signatures within patient cohorts.[8] In a study using single-cell sequencing data, UMAP provided overall faster run times, higher reproducibility, and more informative cluster organization compared to five other techniques, including PCA and t-SNE.[9]

To qualitatively compare the UMAP projection with other dimension reduction algorithms, we show in Figure 1 the projections of (a) principal component analysis[3] (PCA), (b) Isomap,[10] (c) t-stochastic neighborhood embedding[4] (t-SNE), and (d) self-organizing map[11] (SOM). For UMAP,[12] PCA,[13] Isomap,[14] and t-SNE,[15] the default hyperparameter settings of the respective implementations in the R programming language[13] were used. For SOM,[16] a 10x10 grid with hexagonal topology was chosen; otherwise, the package defaults were kept. Figure 1 shows that all techniques resulted in projections in which the mass of the points of the five clusters was preserved. Principal component analysis[3] and Isomap[10] tend to focus on highlighting outliers, as patients with more extreme values are positioned farther away, at the cost that the majority of points is clumped together. The projection of t-SNE is similar to the one UMAP. As SOM produces a discrete output space, distance relationship between neighboring units is unclear.

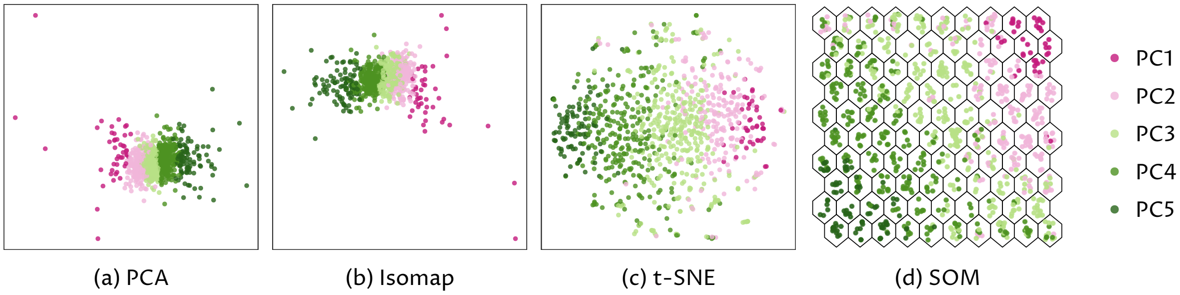

Figure 1: **2D projections of other widely used dimension reduction algorithms ($\Delta(t_0, t_1)$ data)** (a) Principal component analysis[3] (PCA), (b) Isomap,[10] (c) t-stochastic neighborhood embedding[4] (t-SNE), (d) self-organizing map[11] (SOM). Point colors are according to pathways of change (PC).

References

[1] Niemann, U. *et al.* Phenotyping chronic tinnitus patients using self-report questionnaire data: cluster analysis and visual comparison. *Scientific Reports* **10**, 1–10 (2020). URL `https://doi.org/10.1038/s41598-020-73402-8`.

[2] Goebel, G. & Hiller, W. *Tinnitus-Fragebogen (TF). Ein Instrument zur Erfassung von Belastung und Schweregrad bei Tinnitus* (Hogrefe, 1998).

[3] Hotelling, H. Analysis of a complex of statistical variables into principal components. *Journal of Educational Psychology* **24**, 417 (1933).

[4] Maaten, L. v. d. & Hinton, G. Visualizing Data using t-SNE. *Journal of Machine Learning Research* **9**, 2579–2605 (2008). URL: `https://www.jmlr.org/papers/v9/vandermaaten08a.html`.

[5] McInnes, L., Healy, J. & Melville, J. UMAP: Uniform manifold approximation and projection for dimension reduction (2020). `https://arxiv.org/abs/1802.03426`.

[6] Diaz-Papkovich, A., Anderson-Trocmé, L. & Gravel, S. A review of UMAP in population genetics. *Journal of Human Genetics* **66**, 85–91 (2021).

[7] Diaz-Papkovich, A., Anderson-Trocmé, L., Ben-Eghan, C. & Gravel, S. UMAP reveals cryptic population structure and phenotype heterogeneity in large genomic cohorts. *PLoS genetics* **15**, e1008432 (2019).

[8] Granja, J. M. *et al.* Single-cell multiomic analysis identifies regulatory programs in mixed-phenotype acute leukemia. *Nature Biotechnology* **37**, 1458–1465 (2019).

[9] Becht, E. *et al.* Dimensionality reduction for visualizing single-cell data using UMAP. *Nature Biotechnology* **37**, 38–44 (2019).

[10] Tenenbaum, J. B., De Silva, V. & Langford, J. C. A Global Geometric Framework for Nonlinear Dimensionality Reduction. *Science* **290**, 2319–2323 (2000).

[11] Kohonen, T. *Self-Organizing Maps* (Springer Berlin Heidelberg, 1995).

[12] Konopka, T. *umap: Uniform Manifold Approximation and Projection* (2020). URL `https://CRAN.R-project.org/package=umap`. R package version 0.2.7.0.

[13] R Core Team. *R: A Language and Environment for Statistical Computing.* R Foundation for Statistical Computing, Vienna, Austria (2022). URL `https://www.R-project.org/`.

[14] Kraemer, G., Reichstein, M. & Miguel Mahecha, D. dimRed and coRanking—Unifying Dimensionality Reduction in R. *The R Journal* **10**, 342–358 (2018). URL `https://journal.r-project.org/archive/2018/RJ-2018-039/index.html`. CoRanking version 0.2.3.

[15] Krijthe, J. H. *Rtsne: T-Distributed Stochastic Neighbor Embedding using Barnes-Hut Implementation* (2015). URL `https://github.com/jkrijthe/Rtsne`. R package version 0.15.

[16] Wehrens, R. & Buydens, L. M. C. Self- and Super-Organizing Maps in R: The kohonen Package. *Journal of Statistical Software* **21**, 1–19 (2007).
